# Comparative Genomics Reveals the High Copy Number Variation of a Retro Transposon in Different *Magnaporthe* Isolates

**DOI:** 10.3389/fmicb.2019.00966

**Published:** 2019-05-06

**Authors:** Pankaj Kumar Singh, Ajay Kumar Mahato, Priyanka Jain, Rajeev Rathour, Vinay Sharma, Tilak Raj Sharma

**Affiliations:** ^1^Indian Council of Agricultural Research (ICAR)-National Research Centre on Plant Biotechnology, New Delhi, India; ^2^Department of Bioscience and Biotechnology, Banasthali University, Tonk, India; ^3^Department of Agricultural Biotechnology, Chaudhary Sarwan Kumar Himachal Pradesh Krishi Vishvavidyalaya (CSK HPKV), Palampur, India; ^4^National Agri-Food Biotechnology Institute, Mohali, India

**Keywords:** *Magnaporthe*, genome, SINE, effector, repeat, RP-2421, RML-29

## Abstract

*Magnaporthe oryzae* is one of the fungal pathogens of rice which results in heavy yield losses worldwide. Understanding the genomic structure of *M. oryzae* is essential for appropriate deployment of the blast resistance in rice crop improvement programs. In this study we sequenced two *M. oryzae* isolates, RML-29 (avirulent) and RP-2421 (highly virulent) and performed comparative study along with three publically available genomes of 70-15, P131, and Y34. We identified several candidate effectors (>600) and isolate specific sequences from RML-29 and RP-2421, while a core set of 10013 single copy orthologs were found among the isolates. Pan-genome analysis showed extensive presence and absence variations (PAVs). We identified isolate-specific genes across 12 isolates using the pan-genome information. Repeat analysis was separately performed for each of the 15 isolates. This analysis revealed ∼25 times higher copy number of short interspersed nuclear elements (SINE) in virulent than avirulent isolate. We conclude that the extensive PAVs and occurrence of SINE throughout the genome could be one of the major mechanisms by which pathogenic variability is emerging in *M. oryzae* isolates. The knowledge gained in this comparative genome study can provide understandings about the fungal genome variations in different hosts and environmental conditions, and it will provide resources to effectively manage this important disease of rice.

## Introduction

Rice is one of the most important food security crops of developing countries. It has been reported that more than one-third of the world’s population depend on it for their major calorific intake (Goff, 1999). The most devastating and economically significant disease that affects rice crops is a rice blast disease, produced by the fungal pathogen *Magnaporthe oryzae*. This disease has been a major problem in the most rice-producing areas and causes substantially huge yield losses of up to 30% in all parts of the world, under favorable environmental conditions (Talbot, 2003; Skamnioti and Gurr, 2009). This disease can be effectively managed by the use of fungicides, however, use of resistant varieties is the best option in the current scenario, which does not have any harmful effect on the environment (Singh et al., 2018a). Nonetheless, newly developed resistant rice cultivars are often defeated against the fungus after only a few years in the field due to the highly variable nature of the pathogen (Sharma et al., 2012). Blast disease resistance in rice is contingent on the existence of a related cognate avirulence (*Avr*) gene in *M. oryzae* and follows a typical gene-for-gene hypothesis (Flor, 1971; Valent, 1990).

Identification of new *Avr* genes and effector molecules from *M. oryzae* can be useful to understand the molecular mechanism involved in the fast evolution of different races of this fungus. It may help to reduce the occurrence of quick breakdown of blast resistance by the identification of potential *R* genes and further its application in rice resistance breeding program (Singh et al., 2018b). The emergence of advanced virulent strains of the plant pathogen is governed by *Avr* gene alternations through frame-shift mutations (Ridout et al., 2006), point mutation (Joosten et al., 1994), deletions (Dodds et al., 2006), and insertion of transposons (Kang, 2001; Kang et al., 2001). Instability of *Avr* genes via these genetic changes is a common process of evolution toward virulence as revealed by the extensive surveys of large natural populations of fungal pathogens (Van de Wouw et al., 2010). So far, over 40 *Avr* genes have been identified from *M. oryzae* (Valent and Khang, 2010), of which 11 genes, *PWL1* (Kang et al., 1995), *PWL2* (Sweigard et al., 1995), *AvrPita* (Orbach et al., 2000) ACE1 (Fudal et al., 2005), *Avr1CO39* (Leong, 2008), *AvrPiz-t* (Li W. et al., 2009), *AvrPia*, *AvrPii*, *AvrPik/km/kp* (Yoshida et al., 2009), *AvrPi9* (Wu et al., 2015), *AvrPib* (Zhang et al., 2015), and *AvrPi54* (Ray et al., 2016) have been cloned and characterized. It is very essential to identify the effectors and *Avr* genes in the field isolates of *M. oryzae* in different geographical regions of the world, which may help in deployment of *R* genes with durable resistance against *M. oryzae*.

Many fungal genomes, including those from pathogenic fungi, have been decoded and made available in the public domain (Galagan et al., 2005). These provides ample opportunity for future investigations into the processes of host-pathogen interactions at the molecular level. Such studies advance our understanding about the evolution of fungal virulence genes and to recognize the gene subsets that are responsible for pathogenicity in the fungi (Soanes et al., 2002; Veneault-Fourrey and Talbot, 2005). Among the plant pathogenic fungi, *M. oryzae* (70-15 strain) was the first genome completely decoded in 2005 by Sanger sequencing method (Dean et al., 2005). Subsequently, several genomes of *M. oryzae* have been re-sequenced from various isolates of the fungus using next generation sequencing (NGS) approaches. Field isolates from China (Y34) and Japan (Ina168 and P131) were sequenced applying 454 Roche platform (Yoshida et al., 2009; Xue et al., 2012). Various field isolates from China and India, FJ81278, HN19311, B157, and MG01 have been sequenced using Illumina NGS technology (Chen et al., 2013; Gowda et al., 2015). Interestingly, the whole genome decoding of various isolates has disclosed exceeding a mega-base pair isolate specific sequences, which contain hundreds of isolate specific genes. These genes determine the specificity of a particular isolate and this specificity might be due to the racial evolution after a long period of time and host range specificity, chromosomal variation and variability in repetitive elements (Dean et al., 2005; Yoshida et al., 2009; Chen et al., 2013). Several lineages of *Magnaporthe* are known to infect a range of host plants throughout the world (Gowda et al., 2015) and obviously they have their own specificity at the genome level. Additional comparative genome study with previously sequenced genomes help us to identify novel avirulence and effector molecules and the mechanism of host-pathogen interaction.

Several reports have been published that the transposable repeat elements act a key role in the evolution of *M. oryzae* genome and subsequently affect the virulence spectrum of the fungus (Xue et al., 2012; Chen et al., 2013; Gowda et al., 2015). A transposable element (TE) Pot3 is shown linkage with the virulence spectrum of *M. oryzae* strains (Kang et al., 2001; Li W. et al., 2009; Dai et al., 2010; Singh et al., 2014). Although, mechanisms underlying the variability of *Avr* determinants in *M. oryzae* is largely unknown. Insertion of TE (Pot3) within the promoter region of the *Avr g*ene (*AvrPita* and *AvrPiz-t*) becomes a major causal event for the gain of virulence in the mutant strains of *M. oryzae* (Kang et al., 2001; Li W. et al., 2009; Singh et al., 2014). It is compelling that transposable element (TE) was always found to be connected with the malfunction or rearrangement of *M. oryzae Avr* genes, *AvrPita*, *AvrPiz-t*, *Avr1CO39*, and *ACE1* (Kang et al., 2001; Farman et al., 2002; Bohnert et al., 2004; Li W. et al., 2009; Singh et al., 2014). Short interspersed nuclear element (SINE) is a retro transposable repeat element and is reported for to be actively involved in insertional inactivation of genes (Wallace et al., 1991; Goldberg et al., 1993) and in the formation of chimeric sequences (Daniels and Deininger, 1991). Such insertional inactivation of genes are operated by the mechanisms of mRNA truncation, altered polyadenylation, and modified protein structure (Oliviero and Monaci, 1988; Vidal et al., 1993; White, 1994). These repeat elements can also contribute to genomic flux and may even result in genetic disorders through the homologous and nonhomologous recombination events (Lehrman et al., 1987). Keeping in perspective the significance of *R-Avr* gene interaction in rice-*M. oryzae* pathosystem and the importance of repeat elements in *M. oryzae* genome, the present investigation was planned with the objectives of (i) decoding the genomes of most virulent and a least virulent strain of *M. oryzae*, (ii) identification of structural features in these two genomes, and (iii) comparative analysis of different *M. oryzae* genomes.

## Materials and Methods

### *Magnaporthe genome* Sequencing and Assembly

*Magnaporthe oryzae* isolate Mo-nwi-31 (RP-2421) was selected for whole genome sequencing. The whole genome sequence of another isolate Mo-nwi-55 (RML-29) was generated by using Roche 454 FLX Titanium (Roche Diagnostics Corporation, Rotkreuz, Switzerland) sequencing machine. Although this genome was previously assembled and a part of data has been published by Dr. T. R. Sharma’s group (Accession no. AZSW00000000; Ray et al., 2016). Both RML-29 and RP-2421 cultures were collected from the North West Himalayan region of India. The former is found to be avirulent on most of the differential rice blast resistance monogenic lines while the latter showed virulent symptoms of rice blast disease on most of the lines. These differential lines were developed and provided by the International Rice Research Institute (IRRI), Philippines. RP-2421 genome sequencing was performed by Illumina HiSeq-1000 machine at the National Research Centre on Plant Biotechnology, New Delhi. Read quality and trimming of adapter sequences from the reads were performed by three softwares, CLC Genomics Workbench 6.5 (CLC Bio, Aarhus, Denmark), FastQC V0.10.1^1^ and NGSToolKit (Patel and Jain, 2012). The filtered reads of both data generated by 454 Roche and HiSeq-1000 were utilized for *de novo* assembly using CLC Genomics Workbench 6.5 (CLC Bio, Aarhus, Denmark) on Fijutsu work station with a capacity of 192 Gb physical memory. The contigs obtained after the *de novo* assembly was subjected to reference based assembly using reference genome of *M. oryzae* isolate 70-15 (version 8, Broad Institute, Harvard). All the unmapped contigs (or unique contigs) were used as query in matching with unplaced raw reads generated during the reference genome sequencing^2^ in the BLASTN programme of ncbi-blast-2.2.28+ package^3^ for checking their magnitude of uniqueness exiting in the RML-29 and RP-2421 genomes. Two additional genomes of P131 (Accession no. AHZT00000000) and Y34 (Accession no. AHZS00000000) were retrieved from NCBI genome databank^4^ and assembled them by mapping with the reference sequences of 70-15. The contigs of RP-2421 genome generated in *de novo* assembly were submitted at the NCBI genome databank. The assembled chromosome specific pseudo molecules of both genomes RML-29 and RP-2421 were used for further structural and functional annotations, and comparative genome analysis.

### PCR Amplification From Isolate Specific Regions

Isolate specific primers were designed using template of isolate specific sequences from each RML-29 and RP-2421 strains (Supplementary Table 1). Primer3 software was used for the primer designing (Untergasser et al., 2012). PCR (G-storm thermo cycler, Somerton Biotechnology Centre, Somerton, United Kingdom) amplification was performed to confirm the isolate specific sequences present in the both strains, RML-29 and RP-2421 using standard PCR protocol of TaKaRa Taq^TM^ DNA polymerase (DSS Takara Bio India Pvt. Ltd.) with a fixed 55°C annealing temperature for all the primer pairs.

### Pan-Genome Analysis

Total 10 *M. oryzae* assembled genome sequences and their raw sequencing reads were retrieved from the NCBI genome database on the basis of N50 value and genome coverage criteria. Genome accession numbers and SRA accessions are given in Supplementary Table 2. The SRA data of each genome was downloaded using prefetch module of NCBI SRAToolkit^5^ and converted into fastq format by fastq-dump module of the toolkit. The pan – genome was constructed with the help of ppsPCP software (Qamar et al., 2019). The presence and absence variation (PAV) analysis was also executed with this software with default parameters. Before this analysis, total genes were predicted from each isolate by Augustus (Stanke et al., 2006) using *Magnaporthe* trained dataset. For further analysis, raw reads were aligned against the pan-genome sequences using GraphMap (Sovic et al., 2016) and aligned sam files were converted and sorted into bam format using samtools (Li H. et al., 2009).

### Repeat Identification in *Magnaporthe* Genomes

Assembled sequences of both the genomes RML-29 and RP-2421 were passed through RepeatModeler 1.0.8^6^ for the *de novo* repeat group discovery. In this *de novo* analysis, Repeat Scout, a module of RepeatModeler software was used to identify a set of repeat elements that was further utilized by Recon, another module of the same software to generate a classified consensus repeat library. The consensus library was then merged further with custom library prepared by using some major repeat classes *viz*., Grasshopper, Maggy, MGL, Mg-MINE, SINE, Pot2, MGRL-3, Mg-SINE, Ch-SINE, Occan, Pot3, Pyret, RETRO 5, RETRO 6, RETRO 7, and Cluster 1-9. The merged repeat library was subjected to RepeatMasker 4.0^7^ for homology based masking of the repeat regions in targeted *Magnaporthe oryzae* genome sequences.

### Analysis of Structural Variants

Structural variants such as single nucleotide polymorphic sites (SNPs), Insertion-Deletion (InDel), and genome duplications were performed by MUMmer 3.23 (Kurtz et al., 2004), Samtools^8^ and CLC Genomics Workbench 6.5 (CLC Bio, Aarhus, Denmark). Default parameters were kept for all these analyses.

### Gene Prediction and Functional Genome Annotation

Gene was predicted for each genome separately using the masked resulted sequences in the RepeatMasker analysis. The gene prediction was carried out using FGENESH 2.6 module of Molquest^9^. The prediction was based on the trained dataset of *Magnaporthe* available with the FGENESH module. The predicted genes were used for gene ontology and assigned their respective functional categories. Transfer RNA-coding regions were searched using tRNAscan-SE (Lowe and Eddy, 1997).

### Secretome Analysis

Secretory proteins were identified by a pipeline developed in this study. All the proteins of a genome were initially subjected to SignalP 4.1 (Petersen et al., 2011) and TargetP 1.1 (Emanuelsson et al., 2007) analysis. Results of both TargetP (Loc = S) and SignalP (D-score = Y) analyses were combined and then passed to TMHMM 2.0^10^ for scanning transmembrane spanning regions in these proteins. All proteins with 0 TMs or 1 TM predicted through TMHMM if located in the first 40 amino acids were kept for further analysis. All GPI-anchor proteins were identified by the big-PI^11^. At this step, all proteins resulted from TMHMM and big-PI were also used to predict their localization within the cell by ProtComp 9.0 using the LocDB and PotLocDB databases^12^. Finally, refined secreted proteins were analyzed by running “runWolfPsortSummary fungi” of WoLF PSORT 0.2 (Horton et al., 2007). After getting refined secreted proteins, the functional annotation of these refined proteins was performed via Interproscan 5-RC-7 (Zdobnov and Apweiler, 2001). All the analyses were performed with default parameters.

### Gene Ontology and Gene Function Categorization

Gene ontology and its functional categorization were performed by using a set of softwares, BLASTX (Altschul et al., 1990), Interproscan 5-RC-7 (Zdobnov and Apweiler, 2001), and Blast2Go (Conesa et al., 2005). Top 10 hits of BLASTX results and Interproscan results in xml format were supplied for Blast2Go analysis. The obtained results of Blast2GO analysis were considered only for biological process category and further summarized them into 11 broader biological sub-processes.

### Comparative Analysis of Five *M. oryzae* Genomes

Five *M. oryzae* genomes were considered for comparative genome analysis. The whole genome sequences of two strains RML-29 and RP-2421 were generated in the current study and genome sequences of two strains P131 and Y34 was downloaded from NCBI^13^ along with the reference genome sequence of the strain 70-15 selected for comparative genome analyses.

### Orthologs Identification and Genome Phylogeny

Orthologous identification in all the five genomes was done using OrthoMCl 1.4 (Li et al., 2003). The single copy ortholog was chosen for phylogenetic analysis. The distance matrix was calculated using YN00 method of PAML 4.8 (Yang, 2007). The tree was generated by the Neighbor Joining method of Phylip 3.695 package (Felsenstein, 2005). The synonymous and nonsynonymous calculation was done with KaKs Calculator (Zhang et al., 2006) and CodelML of PAML 4.8 (Yang, 2007).

The results obtained in various analyses of all the five *Magnaporthe* genomes were visualized using Circos 0.68 (Krzywinski et al., 2009), and SyMap 4.2 (Soderlund et al., 2006).

## Results

### Genome Sequencing, Quality Check, and Assembly

Two strains of *M. oryzae*, RML-29, and RP-2421 were selected for whole genome sequencing project. This selection was based on the virulence spectrum of these isolates on differential blast resistance monogenic rice lines (Supplementary Table 3). RML-29 was found to be avirulent on 19 of total 25 rice blast resistance monogenic lines (Supplementary Table 4). While only 5 of these lines showed resistance reactions to the isolate RP-2421 (Supplementary Table 4). Rice lines Lijiang Xintuan Heigu (LTH) and Taipei-309 were used as susceptible controls in the pathotyping experiments. Based on this study these were characterized as avirulent (RML-29) and virulent (RP-2421) isolates. Whole genome sequencing was performed by using NGS technology. Total 1,429,817 and 452,970,034 raw reads of RML-29 and RP-2421, respectively were subjected for quality check and trimming of bad quality sequences (<20 Phred value). In this quality check, over 92% of high quality (HQ) reads and total 39,430,338,647 HQ bases present in HQ reads were obtained from the RP-2421 genome (Supplementary Table 5). Whereas, 99.99% nucleotides were passed as HQ bases in RML-29.

High quality combined sequences of paired reads for RML-29 and RP-2421 were 542.28 Mb (454 FLX, Roche) and 39.78 Gb (Illumina), representing ∼13.2- and ∼969.7-fold genome coverage, respectively and used for the *de novo* assembly (Table 1). Initially 1,429,684 and 416,663,802 total reads of RML-29 and RP-2421, respectively were taken for the assembly (Table 1 and Supplementary Figures 1A, 2A). In this assembly, 6934 and 13347 total contigs were generated from Roche (RML-29) and Illumina (RP-2421) data, respectively (Table 1 and Supplementary Figures 1B,C, 2B,C). These contigs were mapped back on the reference genome sequence of *M. oryzae* isolate 70-15 (version 8, Magnaporthe Comparative Sequencing Project, Broad Institute of Harvard and MIT). Total 5363 (86.92% of total bases) and 5280 (90.69% of total bases) contigs of RML-29 and RP-2421, respectively were mapped to the reference sequences (Table 1 and Supplementary Table 6). The N50 and largest contig’s size of RML-29 were 10.40 and 86.48 kb, respectively, while these values were 35.35 and 239.05 kb obtained in RP-2421 (Table 1). Average contig’s size was higher in RML-29 than that of RP-2421 (Table 1). However, 2.03 Mb (13.08% of total bases) and 4.46 Mb (9.31% of total bases) regions of RML-29 and RP-2421, respectively were not mapped to the reference genome (Table 1 and Supplementary Table 6). These un-mapped regions were called as unique or isolate specific sequences of corresponding *M. oryzae* isolate. Assembled sequences of RP-2421 and RML-29 genomes were submitted in the genome databank of NCBI^14^. These *M. oryzae* isolates were also deposited at Microbial Type Culture Collection and Gene Bank (MTCC), Institute of Microbial Technology (IMTECH), Chandigarh (Supplementary Table 7).

**Table 1:** Summary of RML-29 and RP-2421 genomes’ assemblies.

Features	RML-29	RP-2421
Sequencing reads	1,429,684	416,663,802
Length of sequence read (Mb)	542.28	39,785.67
Coverage of the genome (fold)	∼13.2	∼969.7
Number of contigs	6934	13,347
Number of mapped contigs	5363	5280
Mapped region (Mb)	40.14	40.37
Largest contig’s size (kb)	86.48	239.05
G+C content (%)	51.6	51.0
Average contig’s size (kb)	5.38	3.04
N50 contig’s size (kb)	10.40	35.35
Number of un-mapped contigs ( ≥ 200 bp)	1571	8067
Un-mapped contig’s size (Mb)	2.03	4.46
Genome size (Mb)	42.20	44.85


### Pan-Genome Construction and Presence and Absence Variations (PAVs) Analysis

A total 54 Mb sized pan-genome was constructed using whole genome sequences of 12 different *Magnaporthe* isolates. This *Magnaporthe* pan-genome contained 15287 genes. The PAV events analysis in all the isolates showed that ASM80585v1 and FJ81278 contained maximum and minimum PAVs, respectively (Table 2). BR32 contributed 4.44 Mb consensus sequences to the pan-genome in the form of 970 PAVs, which was the longest isolate-specific region among the isolates. This isolate also had the largest number of isolate-specific genes (Table 2). Only three isolates, RP-2421, BTGP6F, and ASM80585v1 have more than 200 isolate-specific genes, while the average number of isolated-specific genes were 167.33. Average length of PAVs ranged from 0.84 kb (in RP-2421) to 4.65 kb (in BTMP13_1). Overall average length of PAVs was 2.68 kb. The maximum number of isolate-specific genes were obtained in BR32, while the least number was found in US71. The isolate-specific gene density (genes per Mb) was maximum (1041.67) in RP-2421 which was very high compared to the isolates, where as other three isolates, FJ81278, RML-24, and US71 had over 200 gene density. Hence, BTJP4_1 had only 90.94 isolate specific gene density that was the lowest among the isolates.

**Table 2:** List of presence and absence variations (PAVs) in different *Magnaporthe* isolates.

Isolate	Genome size (Mb)	Total PAVs^∗^	PAVs added to pan-genome	Average length of PAVs (kb)	Isolate-specific region (Mb)	Isolate-specific genes
ASM80585v1	42.31	1626	534	2.36	1.26	226
BR32	44.51	1377	970	4.58	4.44	554
BTGP1b	46.42	1331	275	3.53	0.97	169
BTGP6F	45.67	1395	508	3.74	1.90	276
BTJP4_1	41.47	436	166	3.26	0.54	49
BTMP13_1	43.86	969	453	4.65	2.10	193
CD156	43.98	526	112	2.32	0.26	36
FJ81278	37.87	197	99	1.26	0.12	31
FR13	44.41	526	85	2.25	0.19	36
RML-24	42.20	447	120	1.40	0.16	44
RP-2421	44.85	493	438	0.84	0.36	375
US71	44.23	478	50	1.93	0.09	19


*^∗^PAVs identified in respective isolate against the Magnaporthe pan-genome.*

### Synteny Analysis of Virulent and Avirulent Strains

**FIGURE 1:**
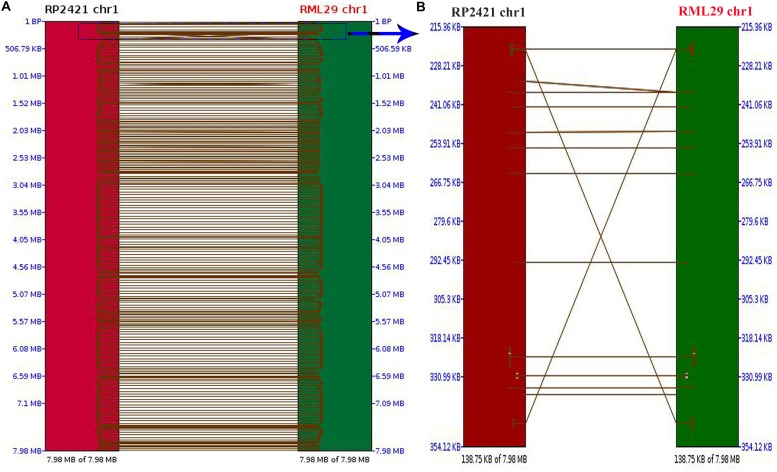
Pairwise comparison between chromosome 1 of RML-29 and RP-2421. **(A)** Showing matching regions of the chromosome in both genomes and **(B)** displaying zoomed view of a selected region within the chromosome marked with blue colored rectangle.

Comparison between the mapped regions of RML-29 and RP-2421 was performed to find the order of short nucleotide sequences and level of similarity between them. A dot plot of all the 8 chromosomes was compared between RML-29 and RP-2421 genomes (Supplementary Figure 3). 1129 total hits were obtained in this synteny analysis, and Pearson correlation coefficient (Pearson R) values for all the hits were found greater than 0.99. Pearson R measures linear association between two variables ranges from +1 to -1. Out of 1129, 249 hits were matched to the chromosome 1 and two short regions 138.75 kb of the chromosomes from RP-2421 and RML-29, respectively were separately aligned to see a clear view of the regions with matching DNA fragments of the first genome to the second genome along with the matched DNA fragment orders positioned in the aligned region (Figure 1). However, the rest of the chromosome 2, 3, 4, 5, 6, 7, and 8 generated 209, 175, 158, 104, 109, 81, and 44 hits, respectively between the genomes.

### SNPs and InDels Analyses of *M. oryzae* Genomes

**FIGURE 2:**
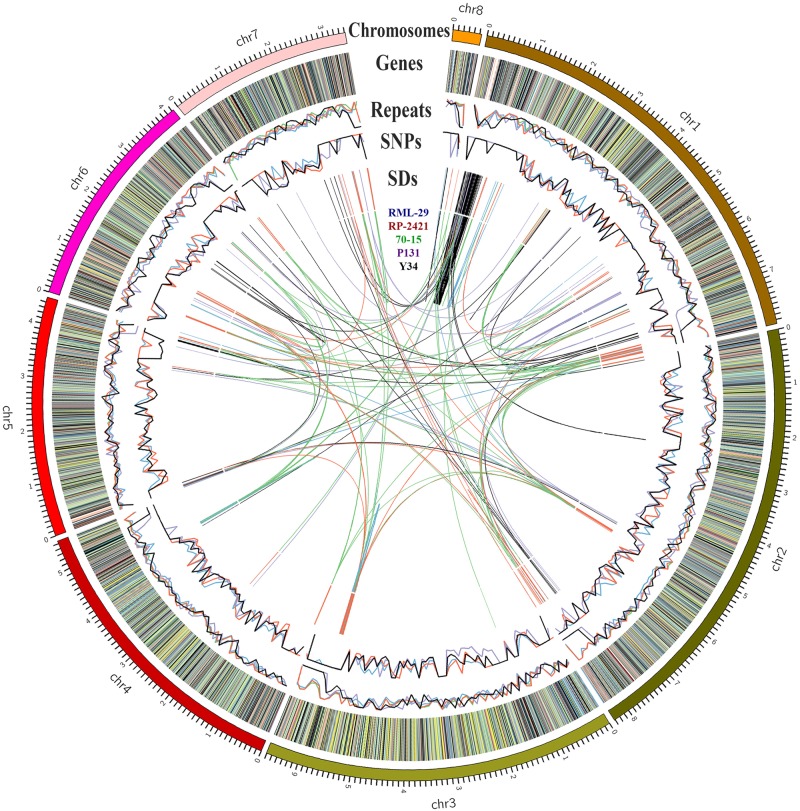
Genome organization of 70-15, RP-2421, RML-29, P131, and Y34. Size of each chromosome marked in Mb. Genes in RP-2421 categorized by gene function along the chromosomes: very deep green, growth, and development; brown, hypothetical; orange, miscellaneous; blue, nucleic acid metabolism; very deep red, pathogenesis; gray, physiological; yellow, protein synthesis; very light red, repeat elements; purple, response to stress; very light green, transportation, and signaling; black, unknown. Repeats and SNPs denote in percentage and in 100 kb windows, respectively. SDs means the segmental duplications present intra- and inter-genome level.

Structural variations were determined in both RML-29 and RP-2421 genomes by comparing their sequences with the reference genome separately. In both the genomes 29776 and 35332 single nucleotide variations (SNVs) were identified from RML-29 and RP-2421, respectively (Supplementary Table 8 and Figure 2). Insertions-deletions (InDels) and replacement events were found 4.4 and 1.2 times higher in RML-29 than RP-2421, respectively (Supplementary Table 8). The multi nucleotide variations (MNVs) were obtained more in RP-2421 than RML-29. SNVs were found more in all the chromosomes of RP-2421 than RML-29, except chromosome no. 1 (Supplementary Table 8).

### Repeat Element Analysis

Repeat elements were identified from both RML-29 and RP-2421 genomes. Total 75 and 94 classified consensus repeat sequences obtained in the *de novo* analysis were used for custom library preparation of RML-29 and RP-2421, respectively. The custom libraries were further exploited to search homology based repeat elements from these strains. Cluster 1–9 repeat elements were considered to include in this analysis by following a previous study (Xue et al., 2012). The nucleotide sequences 1579 (total size = 43,037,792 bp) and 8075 (total size = 45,510,614 bp) of both RML-29 and RP-2421 isolates, respectively were subjected for homology based repeat identification. Similarly, all other isolates included in the pan-genome analysis were also subjected for the repeat masking in the genome sequences (Table 3). The highest level of repeats were masked in BTGP1b genome (18.50% of the genome), whereas the least level were detected in P131 with 3.15% of the genome. The maximum copy number of SINE was found in RP-2421 (2029), which was very high in all the 15 isolates (Table 3). Total nucleotide bases excluding “N” were counted as 42,186,663 and 45,510,614 bp for RML-29 and RP-2421, respectively. Total 11.78% and 12.28% sequences were masked in this analysis for RML-29 and RP-2421, respectively. A total of 6995 interspersed repeats was identified in RP-2421, while a lower number of the repeats 4656 were obtained in RML-29 (Table 4). These repeats covered 10.72 and 11.03% of total sequences of RML-29 and RP-2421 genomes, respectively (Table 4). A sum of 2029 short interspersed repeat elements (SINEs), including 265 unclassified, was detected in RP-2421 strain that was a significantly greater number than that of 79 SINEs recognized from RML-29 (Tables 4, 5). Pot2/Pot4 transposable element showed same frequency in both the genomes, however, a combined copy number of all three RETRO elements were found 1.29 times less in RP-2421 than RML-29 (Table 5). Except Occan and SINE, all other DNA transposon, LTR retrotransposon and LINE were existed in higher copy number in RML-29 than RP-2421 (Table 5). The total frequency of Cluster 1–9 repeats were also found higher in RML-29 than RP-2421 (Table 5). Distribution of repeat elements in both the genomes was following a similar trend across the entire chromosome, except the unique regions of the genomes (Figure 2). Over 6 times more repeat elements were identified from RP-2421 than RML-29 in the unique regions (Supplementary Figure 4).

**Table 3:** Repeat elements and copy number of SINE in different *Magnaporthe* isolates.

Isolate	Genome size (Mb)	Total repeats (%)^∗^	Total interspersed repeats (%)	SINE copy number
ASM80585v1	42.31	9.79	8.38	14
BR32	44.51	13.71	12.72	16
BTGP1b	46.42	18.50	17.61	8
BTGP6F	45.67	16.85	15.89	65
BTJP4_1	41.47	9.19	8.19	11
BTMP13_1	43.86	12.59	11.62	9
CD156	43.98	12.92	11.92	16
FJ81278	37.87	3.87	2.56	114
FR13	44.41	13.31	12.34	17
US71	44.23	13.19	12.20	16
70-15	41.03	12.04	10.82	146
RML-29	42.20	11.78	10.72	79
RP-2421	44.85	12.28	11.03	2029
P131	38.12	3.15	1.90	29
Y34	38.90	3.41	2.15	24


*^∗^Percentage values of respective genome; SINE, Short interspersed nuclear element.*

**Table 4:** Classes of repeat elements identified from *M. oryzae* strains.

Strain	RML-29			RP-2421		
		
Repeat class/family	No. of repeat elements	Length occupied (bp)	% of sequence	No. of repeat elements	Length occupied (bp)	% of sequence
SINEs	79	36,482	0.09	2029	386,407	0.85
LINEs	237	633,539	1.47	960	822,975	1.81
LTR elements	1270	1,676,088	3.89	856	766,986	1.69
DNA transposons	615	713,391	1.66	589	814,939	1.79
Unclassified	2455	1,555,302	3.61	2561	2,228,466	4.89
Interspersed repeats	4656	4,614,802	10.72	6995	5,019,773	11.03
Small RNA	0	0	0.00	83	8591	0.02
Satellites	0	0	0.00	4	1122	0.00
Simple repeats	10,255	392,266	0.91	12787	490,008	1.08
Low complexity	1463	64,596	0.15	1698	75,496	0.17


*LTR, Long terminal repeat; LINE, Long interspersed nuclear element; SINE, Short interspersed nuclear element.*

**Table 5:** Copy number of some major repeat elements identified from *M. oryzae* strains.

Repeat element	RML-29	RP-2421
**DNA TRANSPOSON**		
Pot2/Pot4	8	8
Occan	20	21
Pot3	19	13
**LTR retrotransposon**		
Maggy	2	1
MGLR3	71	45
Pyret	205	151
RETRO5	224	189
RETRO6	80	48
RETRO7	145	110
Grasshopper	51	38
**LINE**		
MGL	56	40
**SINE**		
Mg-SINE/Mg-MINE/Ch-SINE	79	2029
**CLUSTER 1–9 REPEAT ELEMENTS**		
Cluster 1	35	38
Cluster 2	12	11
Cluster 3	44	45
Cluster 4	30	25
Cluster 5	40	25
Cluster 6	30	15
Cluster 7	19	8
Cluster 8	10	29
Cluster 9	5	9


*LTR, Long terminal repeat; LINE, Long interspersed nuclear element; SINE, short interspersed nuclear element.*

Transfer RNA (tRNA) prediction analysis was also conducted in both RML-29 and RP-2421 genomes. A total of 254 tRNA, excluding one undefined was recognized from RML-29, whereas a lower number (203) of this RNA was detected from RP-2421. In addition, 141 pseudo tRNAs were also identified from RP-2421, but none was identified from RML-29.

### Gene Prediction and Identification of Isolate Specific Genes

All the mapped (Chromosome I–VIII) as well as un-mapped (Unique) sequences of both the strains RML-29 and RP-2421 were employed for gene prediction using *M. oryzae* (trained dataset) as reference available in FGENESH software. Gene models 13297 and 13623 were predicted from RML-29 and RP-2421 genomes, respectively. Genes that encoded amino acid (aa) below 50 were excluded from both the genomes. Hence, 12746 and 12957 genes of RML-29 and RP-2421, respectively encoded amino acid equal or above 50 in length were kept for further analyses (Supplementary Table 9). Chromosome wise gene density distributions for RP-2421 genome is given in Figure 2. Average length of protein deduced from RML-29 and RP-2421 were 500.90 and 510.97 aa, respectively. The 6562 and 6586 aa encoding genes were found largest gene of RML-29 and RP-2421, respectively. Isolate specific genes were also predicted from unique sequences of RML-29 and RP-2421 strains (Supplementary Table 9). RP-2421 contained unique sequences over 2 times of RML-29 that yielded isolate specific genes 1.38 times of RML-29 (Supplementary Table 9).

Functional annotations of RML-29 and RP-2421 genomes were performed to assign the function of each gene present in the genomes using a nonredundant (nr) data of NCBI-BLASTX search. Top ten hits of every individual gene were selected for categorization into 11 functional groups based on biological process, namely, growth and development (GD), hypothetical (HY), miscellaneous (MS), nucleic acid metabolism (NM), pathogenesis related (PA), physiological traits (PH), protein synthesis (PS), repeat element related (RE), response to stress (RS), transportation and signaling (TS), and unknown function or no hit found (UN). Approximately 65% of total RML-29 genes were functionally annotated, whereas around 35% of the genes were belonged to HY and UN categories (Supplementary Table 10). However, only 36.65% of isolate specific genes of RML-29 were functionally characterized and rest of 63.35% of the genes is still needed to be characterized and they were fallen into HY and UN groups (Supplementary Table 10). Total 66.42% of genes that predicted from mapped regions of the RML-29 genome, excluding unique genes or isolate specific genes were functionally grouped into all the categories, except HY and UN (Supplementary Table 10). Similarly, 66.49% of total genes, excluding RP-2421 isolate specific genes were found to disperse into all the functional groups, except HY and UN in functional annotation of RP-2421 genome (Supplementary Table 11). Unique genes of RP-2421 were also functionally annotated only 45.26% of the genes and remaining 54.74% were still required to be characterized initially at *in silico* level (Supplementary Table 11).

Automated functional annotation was also accomplished for both RML-29 and RP-2421 genomes using Blast2GO, a web based package. In this process, 6439, 6378, and 3659 genes of RML-29 were functionally annotated into three major gene ontology (GO) groups, molecular function (MF), biological process (BP), and cellular component (CC), respectively (Supplementary Table 12). These major groups, BP, MF, and CC were further descended into 12, 9, and 6 sub-groups, respectively in functional annotation of RML-29 genome (Supplementary Figure 5). Thus, functional annotation based on biological process (BP) accounted 50.04% of total RML-29 genes and maximum contribution in this annotation was of cellular process and followed by a metabolic process (Supplementary Figure 6). Similarly, three main categories of GO, BP, MF, and CC were also divided into 16, 12, and 7 sub-groups, respectively in RP-2421 genome (Supplementary Table 12 and Supplementary Figure 7). With 55.58% of total RP-2421 genes belong to biological process category. Number of genes and their sub-groups categorized on the basis of biological process (BP) present in RP-2421 are given in Supplementary Figure 8.

### Confirmation of Isolate Specific Regions

**FIGURE 3:**
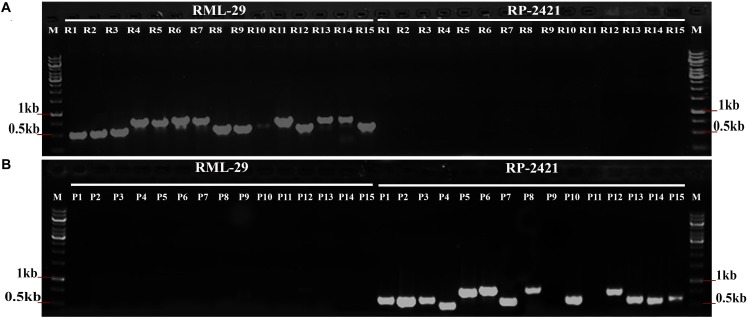
Validation of isolate specific regions in *M. oryzae*. PCR amplification from **(A)** RML-29 and **(B)** RP-2421 using 15 primer sets derived from each strain specific sequences represent strain specificity by presence and absence of bands in the respective strains. M- Molecular weight marker, R1-15 primer sets specific to RML-29, and P1-15 primer sets specific to RP-2421.

PCR amplification was performed using total 30 isolate specific primer pairs, which were specific to RML-29 and RP-2421 (Supplementary Table 1). The specificity of isolate sequences were validated with these primer sets using genomic DNA of both the strains (Figure 3). All the primer sets were worked perfectly as per the expectation since R1-15 primer sets showed bands only with RML-29 genomic DNA and not with RP-2421. Similarly, P1–15 primer sets, except P9 and P11 amplified PCR products only from RP-2421 genomic DNA and not with RML-29. These P9 and P11 primer sets did not show amplification from any of the strains. Approximately 93% of primer sets (28 of 30) were working fine and validated with amplification from genomic DNA of RML-29 and RP-2421.

### Identification of Candidate Effectors From *Magnaporthe oryzae*

**FIGURE 4:**
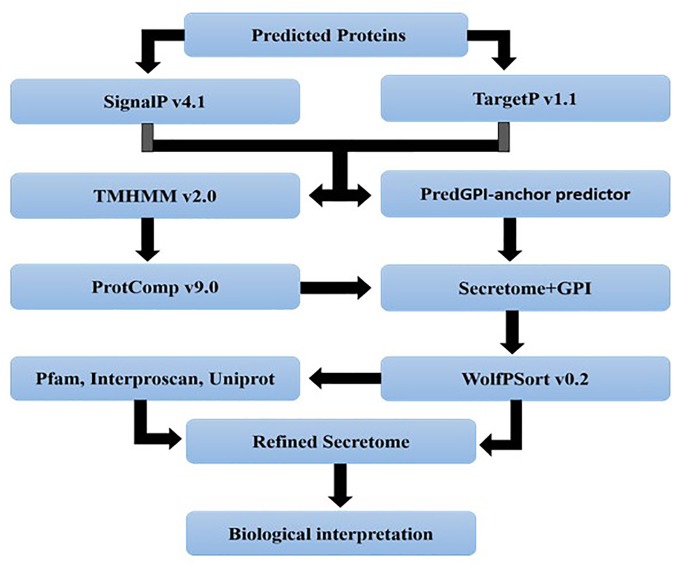
A pipeline showing different steps involved in secretome analysis. The analysis begins with gene prediction, cellular localization, refinement and function assignment to finally result inference. All the steps were performed to analyze the secretome of *M. oryzae* strains.

**FIGURE 5:**
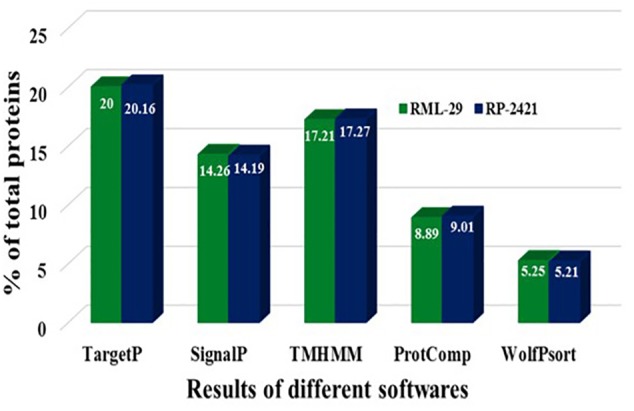
Comparison of proteins resulted in different steps of secretome analysis. Results of different softwares, which were used for secretome analysis are mentioned for RML-29 and RP-2421 strains.

**FIGURE 6:**
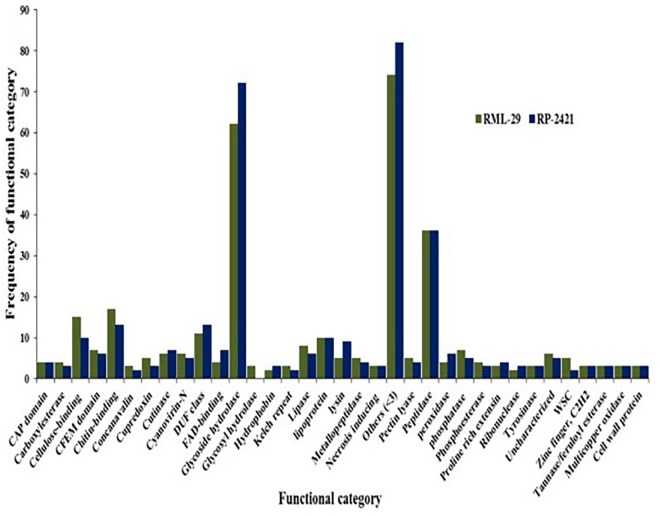
Functional category of secreted protein and their frequencies. Occurrence of secreted proteins with known function in virulent and avirulent isolates of *M. oryzae.*

For the identification of candidate effectors, secretome analysis was performed to discover putative candidate effector molecules from both RML-29 and RP-2421 genomes. To conduct this analysis, a pipeline was developed and used for the identification of secreted proteins from these two genomes (Figure 4). Proteins obtained in each of the different steps of this analysis were almost alike in number in both the genomes (Supplementary Table 13 and Figure 5). This similarity was also followed at the level of functionally categorized genes (Figure 6). Thirty-five categories were made to group all the refined secreted proteins using BLASTP analysis. Based on the examinations of the gene content, 513, and 707 genes, respectively, were distinctive to RML-29 and RP-2421. Overall, 3.70% and 5.52% of these isolate specific genes encoded secreted proteins, and 57.89% and 48.72% of them had no significant homology in the genbank.

### Comparative Analysis of Five *M. oryzae* Genomes

**FIGURE 7:**
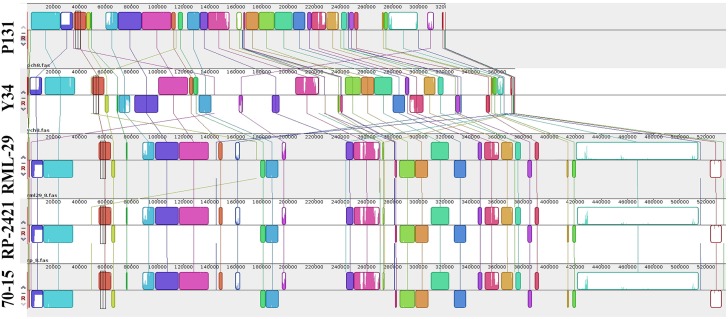
Comparison of the chromosome 8 among five genomes. Illustration of conserved blocks in different colors. Upper and lower blocks at line of respective genome represent positive and negative orientation of DNA fragments present in the chromosome. Colored lines link the blocks and their relations to the chromosome of all the genomes.

For comparative study, three additional genomes of *M. oryzae* strains, 70-15, P131, and Y34 were included along with RML-29 and RP-2421. Assembled sequences of five genomes were compared with Mauve software. Comparisons of all the five genomes at the chromosome level were performed by Symap software. More clear view of the alignments were exported using Mauve software and a representative of these alignments, chromosome VIII (pseudo-chromosome of *M. oryzae*) is given in Figure 7. We found conserved blocks (25040) in this alignment with more than 90% similarity. A progressive alignment guided tree of all the five strains was generated by this analysis (Supplementary Figure 9). RML-29 strain was more closely associated with 70-15 than RP-2421, this grouped into a single cluster; while P131 and Y34 formed a separate group. In the phylogenetic tree, P131 was most divergent among the five strains of *M. oryzae* and 70-15 was least divergent.

The segmental duplication (SD), repeat, SNPs and gene prediction analyses were also performed for all these five genomes (Supplementary Table 14). Density per 100 kb windows of SD, SNPs and repeats were plotted in Figure 2. All the SD containing at least 1000 bp genomic fragment and having more than 90% similarity with different fragment within the genome are given in Supplementary Table 14. Maximum number of SD was obtained from 70-15 (218), followed by RP-2421 (181), RML-29 (153), Y34 (119), and P131 (72). In comparative repeat analysis, occurrence of SINE was estimated for the five isolates and its frequencies were calculated in all the genomes (Supplementary Table 15). None of the isolates, except RP-2421 contained over 150 copies of this retro transposon in their genomes.

**FIGURE 8:**
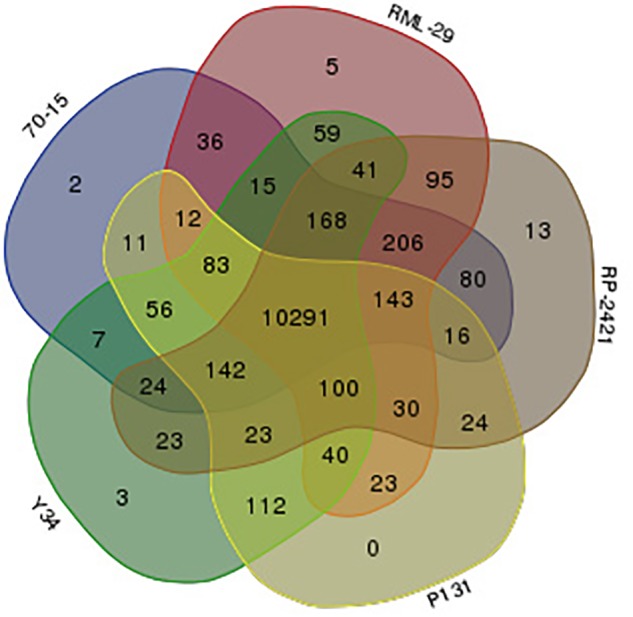
Venn diagram displaying distribution of orthologs clusters made by five *M. oryzae* genomes. Red, gray, blue, yellow, and green colors indicate RML-29, RP-2421, 70-15, P131, and Y34 genomes, respectively. Numbers represent orthologs clusters belonging to corresponding genome(s).

Gene families were identified from these isolates using OrthoMCL program. A sum of combined 61598 proteins, 12746, 12957, 12433, 11672, and 11790 predicted from all the five strains, RML-29, RP-2421, 70-15, P131, and Y34, respectively were subjected to clustering analysis of orthologous groups. In this analysis, 11883 orthologs groups or clusters were formed by using 61598 proteins (Supplementary Table 16). In the five genomes 10291 clusters obtained which were divided into various groups (Supplementary Table 16). Distribution of orthologous clusters was also plotted in Venn diagram and 5, 13, 2, and 3 isolate specific clusters were obtained from RML-29, RP-2421, 70-15, and Y34, respectively (Figure 8). There was no isolate specific cluster in P131 genome (Figure 8). From all the five genomes 10013 orthologous groups formed consisting of five genes in each group (Supplementary Table 16).

### Genome-Wide Evolutionary Analysis

Genome-wide evolutionary study was performed to identify single copy conserved orthologs by analyzing nonsynonymous (Ka) and synonymous (Ks) nucleotide substitutions. In total, 10,013 genes from each of the five genomes were used to identify nucleotide substitutions. All the five genomes RML-29, RP-2421, 70-15, P131, and Y34 were subjected to multiple alignment to make sequence length of same size for further evolutionary study. Estimation of Ka and Ks was performed by YN00 method of PAML package. Total 10 pairwise combinations were made by using all the five genomes (Table 6). RML-29 and RP-2421 genome pair was found to result maximum (10 times to others) value 1.02324 for the Ka/Ks ratio. Maximum (2.18 Million) and minimum (0.51 Million) substitutions, including both Ks and Ka were obtained in 70-15 vs. RML-29 and RML-29 vs. RP-2421 genome pairs, respectively (Table 6). This trend was consistent for divergence time estimation, and the earliest (172,952 years back or 0.173 million years back, MYB) and the latest (0.037 MYB) divergence times among the genome pairs were estimated for the genome pairs of 70-15 vs. RML-29 and RML-29 vs. RP-2421 (Table 6).

**Table 6:** Estimation of divergence time by calculating the synonymous (Ks), nonsynonymous (Ka) and their ratio (Ka/Ks) from five *M. oryzae* strains.

Pairwise strains	70-15 and RML-29	70-15 and RP-2421	70-15 and P131	70-15 and Y34	RML-29 and RP-2421	RML-29 and P131	RML-29 and Y34	RP-2421 and P131	RP-2421 and Y34	P131 and Y34
Method	YN^∗^	YN	YN	YN	YN	YN	YN	YN	YN	YN
Ka	0.173172	0.160275	0.048075	0.099779	0.037219	0.161064	0.110428	0.149676	0.095843	0.081847
Ks	0.172359	0.160587	0.047174	0.099266	0.036374	0.160768	0.109628	0.149827	0.095414	0.081775
Ka/Ks	1.00471	0.99806	1.01909	1.00517	1.02324	1.00184	1.0073	0.998993	1.0045	1.00089
P^∗∗^	1.78E-04	6.25E-01	1.03E-11	4.23E-04	3.38E-13	7.36E-02	3.35E-05	9.95E-01	1.20E-02	5.49E-01
Length	14.14 Mb	14.24 Mb	14.16 Mb	14.19 Mb	14.18 Mb	14.03 Mb	14.10 Mb	14.11 Mb	14.18 Mb	14.14 Mb
Ks-Sites	3.82 M	3.85 M	3.83 M	3.84 M	3.83 M	3.79 M	3.80 M	3.81 M	3.83 M	3.82 M
Ka-Sites	10.30 M	10.40 M	10.30 M	10.40 M	10.30 M	10.20 M	10.30 M	10.30 M	10.40 M	10.30 M
Subs.	2.18 M	2.05 M	0.65 M	1.32 M	0.51 M	2.03 M	1.44 M	1.91 M	1.27 M	1.10 M
Ks-Subs.	0.58 M	0.55 M	0.17 M	0.35 M	0.13 M	0.54 M	0.38 M	0.51 M	0.34 M	0.29 M
Ka-Subs.	1.59 M	1.50 M	0.48 M	0.96 M	0.37 M	1.48 M	1.06 M	1.40 M	0.93 M	0.80 M
D-T (MYB)	0.172952	0.160359	0.047831	0.09964	0.036991	0.160984	0.110213	0.149717	0.095727	0.081828


*^∗^YN00 method of PAML software; ^∗∗^P-value (Fisher); M, Million or Mega; Ks, Synonymous; Ka, nonsynonymous; Subs, Substitutions; D-T, Divergence-Time; MYB, Million years back.*

**FIGURE 9:**
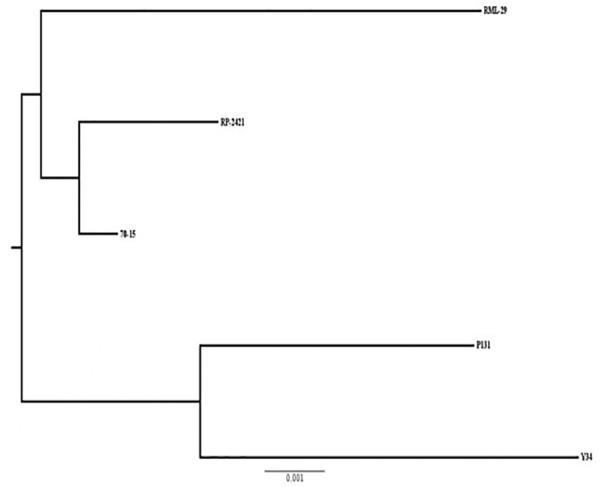
A phylogenetic tree of five *M. oryzae* isolates based on orthologous groups. Relatedness of the isolates based on alignment of 10013 single copy orthologs present in genome wide sequences of the isolates. Scale of substitution rate per site is given at the bottom of the tree.

Phylogenetic relationship based on 10013 conserved orthologs genes was carried out using Phylip software. As expected, RML-29, RP-2421, and 70-15 *M. oryzae* strains formed a separate group and a different cluster was obtained for P131 and Y34 Chinese strains (Figure 9). The strain RP-2421 was more closely related to 70-15 reference strain than RML-29.

## Discussion

In various eukaryotic organisms, comparative study of many genomes of a particular species has been employed to refine the genome assembly, identification and annotation of the genes, and detection of structural variations like repeats, SNPs, InDels, haplotypes, etc. in the genome (Kellis et al., 2003, 2004; Novo et al., 2009; Andersen et al., 2011). *M. oryzae* is well-known for its frequent natural genetic variant occurrence in the field conditions, and the consequence of this is the emergence of new races and disease outbreaks in the rice growing areas of the world (Valent and Chumley, 1991; Talbot, 2003). Comparative genomics analysis of *M. oryzae* also help to understand molecular aspects of the emergence of new virulence mechanism of the pathogen, for proper blast disease control (Farman, 2002; Talbot, 2003). In the present study, two field isolates of *M. oryzae*; RP-2421, and RML-29 from India were compared at the genome level, which were virulent and avirulent, respectively against most of the characterized rice blast resistance genes. Genome analysis of five different *M. oryzae* isolates indicated that the two Indian field isolates (RP-2421 and RML-29) are more closely associated with 70-15 than to the Chinese field isolates (P131 and Y34). However, 70-15 is a laboratory made strain developed from the backcrosses of rice infecting isolate Guy11 with a sibling of a cross related to a nonrice infecting isolate that infects weeping love grass. The overall genome content and composition are similar among these five isolates, but the genome sizes of RP-2421 and RML-29 with only ATGC contents without N’s were slightly higher than that of 70-15. Previously, B157, MG01, P131, and Y34 isolates of *M. oryzae* were also reported to have slightly larger genome size than 70-15 (Xue et al., 2012; Gowda et al., 2015).

The present study revealed that both RML-29 and RP-2421 isolates had some unique isolate specific genomic DNA sequences. The isolates RML-29 and RP-2421 had 513 and 707 unique genes, respectively. While, the pan-genome analysis showed a great reduction in the isolate specific genes in these isolates, because the pan-genome was constructed from 13 different isolates’ information, including reference genome 70-15. RML-29 had only 44 isolates specific genes, however 375 such genes were detected in RP-2421. Interestingly, RP-2421 possessed the highest isolate-specific gene density (1041.67 genes/Mb region, 735 genes in 0.36 Mb region) across the analyzed 12 different *Magnaporthe* isolates. Overall, more than 3% of these unique or isolate specific genes encoded secretory proteins and over 48% of them had no substantial homology in the genbank. These unique genes play diverse roles in the adaptation process of the individual isolate, even so, some of which might perhaps attribute to the specificity of distinct isolates (Xue et al., 2012). Several isolate specific genes in the rice blast fungus have been reported in the previous studies (Yoshida et al., 2009; Xue et al., 2012; Chen et al., 2013; Dong et al., 2015; Gowda et al., 2015), suggesting that the isolate specific genes might be lost or gained during the course of evolution (Xue et al., 2012). Some of the unique genes identified in the present investigation show no hits with the NCBI nonredundant database. Similar results were also found by Xue et al. (2012), and they reported that P131 and Y34 isolate specific genes had no homology with the genes present in the NCBI database. Many authors also claimed that these unique genes might have crucial roles in plant infection process. Moreover, gain or loss of the effector gene is often associated with the unstable telomeric regions of the chromosome (Yoshida et al., 2009). It is thus hypothesized that the presence of isolate specific regions at the chromosomal ends act as a source of new effectors to augment genome evolution of *M. oryzae* (Dong et al., 2015). The presence or absence of avirulence genes may be directly related to the adaptation process of *M. oryzae* (Chuma et al., 2011) and such hypotheses suggest that additional genome decoding of various *M. oryzae* isolates is necessary for characterizing rice infecting strains, as crucial information can be revealed only by surveying outside the “core” genomes (Yoshida et al., 2009). The extensive chromosomal shuffling in asexual reproduction of fungus *Verticillium dahlia* is a general mechanism to make lineage specific regions that supply new effectors for the fungal adaptation (de Jonge et al., 2013). Although sexual reproduction in *M. oryzae* is very rare event reported in the field conditions, but it can be facilitated under laboratory conditions as well (Dong et al., 2015). Such asexually reproducing organisms are often considered to be less flexible in the adaptation process than sexual organisms (Burt, 2000; McDonald and Linde, 2002). The expansion of the genome, possibly signifies a paradigm of evolutionary exchanges, as the price of upholding the additional DNA fragment is compensated by the functional benefits it provides (Raffaele and Kamoun, 2012). However, Dong et al. (2015) hypothesized that horizontal gene transfer (HGT) might be another event that provides the isolate specific genes to operate the evolutionary process in *M. oryzae*. It is known that HGT has a role in the gaining of new genes and functions (Raffaele and Kamoun, 2012).

In the present investigation, the comparative study of five *M. oryzae* isolates; 70-15, RML-29, RP-2421, P131, and Y34, over 100 kb regions scattered in all the 8 chromosomes were found conserved as SD. Duplication is one of the key mechanisms for evolutionary process in the organisms. Xue et al. (2012) also found duplicated sequences in a comparative study of three isolates of *M. oryzae*, 70-15, P131, and Y34 and these sequences seemed to be enriched in the telomeric regions of all the 7 chromosomes. In this study, both inter- and intra-chromosomal duplications were examined, however, higher inter-chromosomal duplications was evident, and only a small part of duplication events were sustained in all the three isolates. They also reported that the duplicated sequences also code for many genes, involved in interactions with the host plant. In the present study a total of 10291 clusters from all the five genomes, 70-15, RML-29, RP-2421, P131, and Y34 were found and these clusters were considered to contain a core gene set for *M. oryzae* genome. The core set of genes obtained from clustering analysis of 61598 genes, found 10013 homologous groups having single copy gene and they are highly conserved across all the five genomes of *M. oryzae*. The cumulative length of all these core genes from all the genomes has potential to estimate the evolutionary forces acted throughout the genome and make them enable to compare with each-other. It is considered that genes having role in the virulence of phytopathogen may be associated with a quick array of evolution for acclimatization to different environments or hosts (Chen et al., 2013). To evaluate gene evolution rate, the nonsynonymous and synonymous (Ka/Ks) ratios of nucleotide substitution in 10013 single copy orthologous genes analysis was performed, in the present study from all the five strains were estimated and found that all the 10 strain pairs, except two are under positive selection pressure. The exceptional pairs 70-15 -RP-2421 and RP-2421 – P131 sustain against the action of resultant purifying force, because the Ka/Ks ratios were less than 1 and very close to 1 in both the pairs, i.e., 0.998060 for 70-15 – RP-2421 and 0.998993 for RP-2421 – P131. The positive evolution pressure was also reported on many genes of several field isolates, FJ81278, HN19311, MG01, and B157 (Chen et al., 2013; Gowda et al., 2015).

Effectors are considered as basic pathogenicity determinants that alter plant innate immunity and assist in disease development during plant-pathogen interactions (Kamoun, 2007). Many *Avr* genes have been earlier cloned and characterized by genetic association analysis, map-based cloning, or loss-of-function approaches (Kang et al., 1995; Sweigard et al., 1995; Orbach et al., 2000; Ahn et al., 2004; Kim et al., 2005; Yoshida et al., 2009). Most of these effectors are small proteins and are secretory in nature. They do not show much homology to known proteins (Ellis et al., 2009; Stergiopoulos and de Wit, 2009; Valent and Khang, 2010). In secretome analyses of the isolates RML-29 and RP-2421 genomes, we obtained 669 and 675 refined candidate effectors, respectively. Of these candidate effectors, approximately 48% of them in both the genomes were not functionally defined and show hypothetical or unknown functions. Diverse nature of characterized effectors is well-known and their least homology with previously identified proteins indicate that further characterization of individual candidate effectors is very essential for molecular study of the host-pathogen interaction.

Since sexual reproduction in *M. oryzae* is a very rare event in natural conditions and genetic variation through asexual reproduction is not possible, still isolate specific genomic sequences are reported in many studies (Dean et al., 2005; Yoshida et al., 2009; Xue et al., 2012; Chen et al., 2013; Dong et al., 2015; Gowda et al., 2015). Translocations of the repeat elements may possibly be one of the key factors for genome variation as well as rapid adaptation of the pathogen to different hosts and environments (Xue et al., 2012). A gain of virulence is often related to the translocation of repetitive elements that generally inactivate or delete PAMP- (pathogen associated molecular pattern) encoding genes whose products trigger the innate plant defense mechanism (Kang et al., 2001; Farman et al., 2002). Thus, understanding the molecular biology of the repetitive elements in the rice blast fungus not only offers an insight into their effects on genome evolution process, but also sheds light on the mechanisms associated with pathogenic variation found at the strain level (Dean et al., 2005). Consistent with this assumption, over 10% of the genomes RML-29 and RP-2421 were covered with repetitive sequences in our study. Similar content of presence of repetitive elements in the genomes of *M. oryzae* were reported in the previous studies as well (Xue et al., 2012; Gowda et al., 2015). However, slightly lower percentage of the repetitive elements was documented in the genomes of 70-15 and 98-06 isolates (Dean et al., 2005; Dong et al., 2015). In present study, the maximum genome sequences masked with repeats was found in BTGP1b (18.50% of genome) and higher percentage of repeats were identified in all the long reads assembled genomes, except BTJP4_1 (BR32, BTGP1b, BTGP6F, BTMP13_1, CD156, FJ81278, FR13, US71) than the short reads assembled genomes (ASM80585v1, FJ81278, P131, Y34, RP-2421, RML-29). One thing is very clearly we observed by our repeat analysis of 15 different Magnaporthe isolates and other published reports (Dean et al., 2005; Xue et al., 2012; Gowda et al., 2015; Dong et al., 2015) that the varying degree of repeat occurrences in the genome of *Magnaporthe* has role in the pathogen survival and in the disease causing ability of the pathogen. Varying copy numbers of different repeats occur in RML-29 and RP-2421 isolates. Surprisingly, the short interspersed nuclear elements (SINEs) show high difference in its copy number distributions between RP-2421 and RML-29. The distribution of this repeat is approximately 25 times more frequent in RP-2421 than that of RML-29. The high copy number of SINEs in *M. oryzae* genome was also reported by Kachroo et al. (1995). They suggested two possible reasons for the high SINE copy numbers in *M. oryzae* genome. One of the reasons is a fusion or insertion of the repeat elements in the genomic region of *M. oryzae* followed by elimination of a large part of the inserted sequence from the genome and second reason is recombination event occurring between the repeat sequence and the genomic sequence of *M. oryzae*. Premature termination of reverse transcriptase could be a main cause for the generation of SINE in *M. oryzae* genome as well as in other eukaryotic genomes (Kachroo et al., 1995). Further, in *Magnaporthe*, SINEs were found to evolve before the divergence of the two host-specific forms, since these are found in both *M. oryzae* and *M. grisea* (Kachroo et al., 1995). The amplification and extensive occurrence of the SINE all over the genome may have foremost impact on structural and functional genomics in *M. oryzae* and could possibly be one of the major mechanisms by which the pathogenic variability and adaptability are generated among the isolates of *M. oryzae* (Kachroo et al., 1995). Similar hypothesis was also given by Xue et al. (2012) that transpositions of repeat elements (TEs) may play an important role in several adaptation processes of the rice blast fungus during host-pathogen interaction, such as transcriptional regulations, duplication of genomic fragments, modulating the genomic content via the addition or deletion of isolate specific sequences.

## Conclusion

Molecular understanding of repeat elements in the rice blast fungus not only offers an insight into their effects on genome evolution process, but also sheds light on the mechanisms of pathogenic adaptation attained by different strains. In the pan-genome analysis, we found several isolate-specific genes (ranging from 19 to 554) identified from each of the 12 different *Magnaporthe* isolates that might be gained or lost during the course of evolution and might have roles in their pathogenicity events and their host-pathogen interactions. RP-2421 possessed the highest isolate-specific gene density (1041.66 genes/Mb region) among the isolates. Higher number of pathogenicity related genes annotated in RP-2421 (154) than RML-29 (145) might justify the correlation between pathogenicity related genes and virulence spectrum of a *M. oryzae* isolate. Also a very high copy number (2029) of short interspersed repeat element (SINE) were found in RP-2421 in comparison with RML-29 (79), and they might have direct correlation with the virulence variability within the isolates. More than six hundreds candidate effectors were identified from both RML-29 (669) and RP-2421 (675) through secretome analysis and most of them (approximately 48%) could not be annotated. For better understanding about the strain specific behavior, in term of pathogenicity, these unknown candidate effectors should be functionally validated. In gene evolution rate analysis, 10013 single copy orthologous genes of five strains, RML-29, RP-2421, 70-15, P131, and Y34 were estimated by pairwise comparison of the strains and we found that all the strain-pair combinations, except two are under positive selection pressure. The resources generated and the knowledge gained through the present comparative study of virulent and avirulent filed isolates of *M. oryzae* can give us understandings about the genome variation process in the fungus under different environmental conditions, and will help in the effective management of rice blast disease.

## Author Contributions

TS conceived and managed the project. PS and TS wrote and revised the manuscript. AM and PJ helped in the data analysis. RR provided fungal cultures. VS gave input in the manuscript preparation. All authors read and approved the final manuscript.

## Conflict of Interest Statement

The authors declare that the research was conducted in the absence of any commercial or financial relationships that could be construed as a potential conflict of interest.

## Footnotes

1 http://www.bioinformatics.babraham.ac.uk/projects/fastqc/

2 ftp://ftp.ncbi.nlm.nih.gov/pub/TraceDB/magnaporthe_grisea

3 http://www.ncbi.nlm.nih.gov/

4 www.ncbi.nlm.nih.gov/genome/

5 https://www.ncbi.nlm.nih.gov/Traces/sra/?view=software

6 http://repeatmasker.org/RepeatModeler.html/

7 http://www.repeatmasker.org/RMDownload.html

8 http://samtools.sourceforge.net/

9 http://www.molquest.com/

10 http://www.cbs.dtu.dk/services/TMHMM/

11 http://mendel.imp.ac.at/gpi/cgi-bin/gpi_pred_fungi.cgi

12 http://www.softberry.com

13 www.ncbi.nlm.nih.gov/genome/

14 https://submit.ncbi.nlm.nih.gov/subs/genome/

## Abbreviations

contig: contiguous
InDel: insertions-deletions
SINE: short interspersed nuclear element
SNP: single nucleotide polymorphism
