# Supplementary material for: Comparative Genomics Reveals the High Copy Number Variation of a Retro Transposon in Different Magnaporthe Isolates
Source: Front Microbiol. 2019 May 6;10:966. doi: 10.3389/fmicb.2019.00966 (PMC6512758; doi:10.3389/fmicb.2019.00966)
Supplement: Supplementary file 1 [file Data_Sheet_1.pdf]

SUPPLEMENTARY TABLE 1 | Primers used in the isolate specific sequence amplifications.

Primer's name	Sequence (5'-3')	Length (nt.)	Tm (°C)	Product size (bp)	Short name
**RML-29 specific primers**					
RM_809_F	TGTTCAAACCTATTAATACCAAAA	24	54.97	719	R1
RM_809_R	TAAGTTAAACAGATGGAAAACAAC	24	54.62		
RM_78_F	TGGAACAGTCTATAGTCCTCAATA	24	54.91	749	R2
RM_78_R	CGCAATAACAAGTAGAAACTCTAA	24	55.08		
RM_1164_F	GCTTTGTAGGAAGAAATAAAAGTC	24	55.05	550	R3
RM_1164_R	AAGATACGAGTCATCAGTTTGTTA	24	54.98		
RM_528_F	CGAGAATAAAGTTACAACGACTTA	24	54.95	517	R4
RM_528_R	CATAATCTGCATAATTTCTGAATC	24	54.94		
RM_1564_F	GTTTGACTGAATCTTTCAAGTG	22	54.18	510	R5
RM_1564_R	AGCTTGTAAAACCAGAAATGAC	22	55.32		
RM_1538_F	GATGATCTTAAGGGCATTAGTC	22	54.71	623	R6
RM_1538_R	ATCATCAAAAGAGGATCCATAG	22	54.94		
RM_1456_F	CTGAACTAGCTAACGAAAAGGT	22	54.88	571	R7
RM_1456_R	TGATTACAGGATTTTGTCTCG	21	55.40		
RM_1313_F	CTAACATATAAGTGTAGCCCGATA	24	54.74	574	R8
RM_1313_R	AGAAACAAACAAAAGACTACAACA	24	55.10		
RM_ 359 _F	TTTGAAGATGTACTCTTTCAGTTG	24	55.55	747	R9
RM_ 359 _R	AAAGTACACTTGTCGTAATTGAAG	24	54.70		
RM_582_F	ACTTTCATTTTCTCCATTATCTTC	24	55.04	764	R10
RM_582_R	AGGTTTAAGTATAGTTCCCTAGCA	24	54.98		
RM_203_F	GGTTTGACATTACTCTAACAACAG	24	54.91	595	R11
RM_203_R	ATAAGATGCATCAACTATCTTCAA	24	54.99		
RM_175_F	CTATACAAATCCTCACACATTCAT	24	55.02	750	R12
RM_175_R	TTTAATTTATTTTTAGCCCGTATT	24	55.04		
RM_1063_F	TATAATCATATTCATCCCAATCAG	24	54.99	732	R13
RM_1063_R	TTTCACCAGATCTTGTTATCTTTA	24	55.05		
RM_348_F	CCTAAGCTTTTCTTAGTCAGAGAT	24	54.83	579	R14
RM_348_R	AATCACGAGTTATTTTAGCTATCC	24	55.25		
RM_988_F	GAGATGATGTTGAGTAGCAAATAG	24	55.05	737	R15
RM_988_R	GTTGTCCCAATCTAAAGGTATAAT	24	54.84		
**RP-2421 specific primer**					
RP_64_F	AGTGGATGTTATTAATGTGATGAG	24	55.02	750	P1
RP_64_R	CCTCTCTGTATTGAGGAGTATCTT	24	55.13		
RP_969_F	ATAACGGAGATTATTACGTAGTCC	24	55.01	511	P2
RP_969_R	ATAATTCTTTTGCAAAGTTAGGTT	24	54.96		
RP_392_F	TTTTTATTGAGAGACAGGTCATTA	24	55.05	607	P3
RP_392_R	TTACACTGCTAACTTTGAGAAGAC	24	54.94		
RP_101_F	GATCGTCAGGTATTACTAGTTGC	23	54.91	585	P4
RP_101_R	CAAATTTATCAGCTAACCATCTAA	24	54.82		
RP_485_F	TTTAATGGAGTTGAATGAGTTCTA	24	55.05	590	P5
RP_485_R	TCGTAGGATTAACATTAATTGAGA	24	55.02		
RP_273_F	GAATTGTGTAAAATCTGTTCAAAG	24	54.97	617	P6
RP_273_R	ATTGTTACACTACAAACAATGACC	24	55.01		
RP_394_F	AAGTAAGAAACCTATTGTTCATCC	24	55.01	788	P7
RP_394_R	TTTACTCGACTGTGTCTGATTACT	24	55.04		
RP_569_F	AATTATTAGACAATCCTAGCGTTC	24	55.25	783	P8
RP_569_R	TTTTCTGTTTACTGCTTCTAACAC	24	54.96		
RP_419_F	TATATGTAACACGAGTGAGGCTAT	24	54.99	567	P9
RP_419_R	GTCTTCTAGACCCTATGGAGATTA	24	55.07		
RP_84_F	CGTACCATTACGATACTAGAACTG	24	55.07	788	P10
RP_84_R	TCTCGTACAGACTGGTCTATTTTA	24	55.14		

**RP_147_F**	**TTTTATTCATACTGTCTGTCGTTT**	**24**	**55.00**	**624**	**P11**
**RP_147_R**	**GAGGAGCCTTAGGTTACATAGTAG**	**24**	**54.97**		
**RP_98_F**	**AGAAATAACACCTTACCCTGTAAC**	**24**	**54.97**	**592**	**P12**
**RP_98_R**	**GAACCAACAGGAGTTTATAGAGTT**	**24**	**55.06**		
**RP_414_F**	**TAAAAGAGGGTAGAAAGAGTTTGT**	**24**	**54.98**	**625**	**P13**
**RP_414_R**	**TGAAACAGCTATTTTTCTGAGTAG**	**24**	**55.04**		
**RP_214_F**	**CTATTTTAATGTTTTCACGTCATC**	**24**	**54.94**	**787**	**P14**
**RP_214_R**	**ATATTAGCAACTGTTCATTCAAAA**	**24**	**55.08**		
**RP_1980_F**	**CACAGACCAGTATTTGACTTTATC**	**24**	**54.83**	**504**	**P15**
**RP_1980_R**	**TGAGTTCTCTTATGATTCAGATGT**	**24**	**54.85**		

SUPPLEMENTARY TABLE 2 | Details of *Magnaporthe* isolates used in pan-genome analysis.

Accession No.	SRA	Isolate	Contigs	N50 (kb)	Size (Mb)	Genome coverage (x)	Sequencing platform
GCA_000805855.1	SRR1561422	ASM80585v1	1161	88.90	42.30	135	Illumina HiSeq
GCA_900474225.1	ERR2612751	BR32	59	4344.89	44.50	97	Nanopore MinION
GCA_900474655.1	ERR2612754	BTGP1b	46	5357.03	46.42	97	Nanopore MinION
GCA_900474175.1	ERR2612755	BTGP6F	84	2015.67	45.67	97	Nanopore MinION
GCA_900474545.1	ERR2612748	BTJP4_1	21	5047.69	41.47	97	Nanopore MinION
GCA_900474475.1	ERR2612753	BTMP13_1	44	4257.48	43.86	97	Nanopore MinION
GCA_900474375.1	ERR2612752	CD156	16	6037.51	43.98	97	Nanopore MinION
GCA_000498135.1	SRR923948	FJ81278	3783	115.75	37.87	34	Illumina GAII
GCA_900474635.1	ERR2612749	FR13	74	2814.03	44.41	97	Nanopore MinION
GCA_900474435.1	ERR2612750	US71	57	3705.38	44.23	97	Nanopore MinION

SUPPLEMENTARY TABLE 3 | Rice blast disease reactions caused by RML-29 and RP-2421 on different rice blast resistance monogenic lines.

**Monogenic line**	***R*-gene**	**Donor cultivar**	**RML-29**	**RP-2421**
IRBL1-CL	*Pi1*	C101LAC	R	S
IRBL3-CP4	*Pi3*	C104PKT	R	R
IRBL5-M	*Pi5*	Moroberekan(RIL249)	R	R
IRBL7-M	*Pi7*	Moroberekan(RIL29)	R	S
IRBL9-W	*Pi9*	WHD-1S-75-1-127	S	S
IRBL11-Zh	*Pi11*	Zhaiyeqing 8	R	S
IRBL12-M	*Pi12*	Moroberekan(RIL10)	R	S
IRBL19-A	*Pi19*	Aichi Asahi	-	S
IRBL20-IR24	*Pi20*	IR24	R	S
IRBLa-A	*Pia*	Aichi Asahi	R	S
IRBLb-B	*Pib*	BL1	R	S
IRBLi-F5	*Pii*	Fujisaki5	R	R
IRBLk-Ka	*Pik*	Kanto51	R	S
IRBLkh-K3	*Pikh*	K3	S	S
IRBLkm-Ts	*Pikm*	Tsuyuake	S	R
IRBLkp-K60	*Pikp*	K60	S	S
IRBLks-F5	*Piks*	Fujisaki5	R	S
IRBLsh-S	*Pish*	Shin2	R	R
IRBLta2-Re	*Pita2*	Reiho	R	S
IRBLz-Fu	*Piz*	Fukunishiki	R	S
IRBLzt-T	*Pizt*	Toride 1	R	S
IRBLt-K59	*Pi-t*	-	S	S
IRBLz^5-CA-1	*Pi-*z^5	-	R	S
IRBLta-CP1	*Pi-ta*	-	R	S
LTH (Lijiang Xintuan Heigu)	-	-	S	S
TP-2	*Pi-54*	Tetep	R	-
Taipei-309	-	-	S	-

- Not tested or no information regarding donor cultivar or not applicable

SUPPLEMENTARY TABLE 4 | Presence of avirulence genes in RML-29 and RP-2421 based on the disease reactions on monogenic rice lines of blast resistance genes.

**Strain**	**Avirulence gene (*Avr* gene)**						**No. of *Avr* gene**
RML-29	*AvrPi-a*	*AvrPi-i*	*AvrPi-ks*	*AvrPi-k*	*AvrPi-z*	*AvrPiz-t*	
	AvrPita	*AvrPi-b*	*AvrPi-sh*	*AvrPi-1*	*AvrPi-3*	*AvrPi-5*	
	AvrPi-7	*AvrPi-12*	*AvrPi-20*	*AvrPi-ta2*	*AvrPi-11*	*AvrPi-2*	
	AvrPi54						19
RP-2421	*AvrPi-sh*	*AvrPi-i*	*AvrPi-3*	*AvrPi-5*	*AvrPi-km*		5

SUPPLEMENTARY TABLE 5 | Summary of quality check statistics for paired end reads of RP-2421 genome.

Particulars	1st-end reads	2nd-end reads
Total number of reads	226,485,017	226,485,017
Total number of high quality (HQ) reads	209,414,634	209,414,634
Percentage of HQ reads	92.46%	92.46%
Total number of bases	22,874,986,717	22,874,986,717
Total number of bases in HQ reads	21,150,878,034	21,150,878,034
Total number of HQ bases in HQ reads	19,329,941,706	20,100,396,941
Percentage of HQ bases in HQ reads	91.39%	95.03%
Number of Primer/Adaptor contaminated HQ reads	100	401
Total number of HQ filtered paired end reads	209,414,133	209,414,133
Percentage of HQ filtered reads	92.46%	92.46%

SUPPLEMENTARY TABLE 6 | Statistics of reference based assembly of *M. oryzae* genomes.

Genome	Particulars	Count	% of reads	Average length	No. of bases	% of bases
**70-15**	Reference sequences	8	-	5,128,466.62	41,027,733	-
**RML-29**	Mapped reads	1,241,806	86.86	379.57	471,355,997	86.92
	Not mapped reads	187,878	13.14	377.54	70,931,139	13.08
	Total reads	1,429,684	100.00	379.31	542,287,136	100.00
**RP-2421**	Mapped reads	378,195,207	90.77	95.40	36,081,578,020	90.69
	Not mapped reads	38,468,595	9.23	96.29	3,704,100,204	9.31
	Total reads	416,663,802	100.00	95.49	39,785,678,224	100.00

- Not applicable

SUPPLEMENTARY TABLE 7 | Submission of assembled whole genome sequences of RML-29 and RP-2421.

Strain	Culture's name	Virulence spectrum	MTCC No.	Locus Tag Prefix	Genome Accession No.	Bioproject No.	Submission Id
**RML-29**	Mo-nwi-55	Avirulent	MTCC11509	Y850	AZSW00000000	PRJNA232298	SUB412044
**RP-2421**	Mo-nwi-31	Virulent	MTCC11506	Y851	JGVY00000000	PRJNA232299	SUB412202

SUPPLEMENTARY TABLE 8 | Structural variants identified from RML-29 and RP-2421.

Genome	RML-29				RP-2421			
**Chromosome**	**SNV**	**MNV**	**InDel**	**Replacement**	**SNV**	**MNV**	**InDel**	**Replacement**
**I**	11145	1679	3915	76	10070	1366	910	75
**II**	3302	364	3829	23	6183	509	859	23
**III**	3796	706	2963	36	4563	683	632	34
**IV**	2000	365	2414	23	2725	341	405	12
**V**	2671	326	2250	17	3746	436	490	11
**VI**	4492	565	1939	48	4907	561	539	33
**VII**	1714	297	1679	14	2077	394	370	8
**VIII**	656	153	222	13	1061	191	117	9
**Total**	29776	4455	19211	250	35332	4481	4322	205

SNV- Single nucleotide variation, MNV- Multiple nucleotide variation, InDel- Insertions and deletions

SUPPLEMENTARY TABLE 9 | Gene predicted from sequences of RML-29 and RP-2421 genomes.

Genome	RML-29		RP-2421	
**Chromosome**	**Size (bp)**	**Gene**	**Size (bp)**	**Gene**
**I**	7975102	2338	7978264	2340
**II**	8315490	2444	8319630	2463
**III**	6602964	2017	6606317	2009
**IV**	5543935	1687	5546721.	1703
**V**	4487536	1342	4489852	1338
**VI**	4131784	1250	4133720	1252
**VII**	3414057	1011	3414625	1000
**VIII**	535571	144	535732	145
**Unique**	2037642	513	4492162	707

SUPPLEMENTARY TABLE 10 | Functional category of genes predicted from RML-29 genome.

Functional category	Chromosome								Unique	Total
	I	II	III	IV	V	VI	VII	VIII		
**GD**	143	147	123	125	119	75	63	8	5	808
**HY**	776	757	639	515	402	402	388	61	241	4181
**MS**	28	20	13	28	17	7	6	0	5	124
**NM**	208	317	301	168	116	102	82	9	16	1319
**PA**	29	23	27	18	18	14	14	1	1	145
**PH**	685	752	428	492	445	396	275	34	125	3632
**PS**	67	61	148	63	33	32	19	1	9	433
**RE**	137	90	82	54	40	66	51	13	10	543
**RS**	15	6	15	11	10	9	10	1	3	80
**TS**	211	240	213	191	128	135	87	10	14	1229
**UN**	39	31	28	22	14	12	16	6	84	252

GD: Growth & development; HY: Hypothetical; MS: Miscellaneous; NM: Nucleic acid metabolism; PA: Pathogenesis related; PH: Physiological traits; PS: Protein synthesis; RE: Repeat element related; RS: Response to stress; TS: Transportation & signaling; UN: Unknown function or no hit found

SUPPLEMENTARY TABLE 11 | Functional category of genes predicted from RP-2421 genome.

Functional category	Chromosome								Unique	Total
	I	II	III	IV	V	VI	VII	VIII		
**GD**	150	151	128	130	121	77	65	8	17	847
**HY**	795	782	630	529	407	390	381	57	245	4216
**MS**	28	21	12	28	16	6	6	0	2	119
**NM**	222	326	313	174	120	106	87	11	28	1387
**PA**	28	23	27	20	18	14	14	1	9	154
**PH**	675	744	425	490	435	403	270	39	178	3659
**PS**	71	61	149	63	32	33	20	1	14	444
**RE**	119	81	70	44	40	67	45	12	13	491
**RS**	15	6	15	11	10	9	11	2	0	79
**TS**	210	248	216	193	127	134	88	10	59	1285
**UN**	27	20	24	21	12	13	13	4	142	276

GD: Growth & development; HY: Hypothetical; MS: Miscellaneous; NM: Nucleic acid metabolism; PA: Pathogenesis related; PH: Physiological traits; PS: Protein synthesis; RE: Repeat element related; RS: Response to stress; TS: Transportation & signaling; UN: Unknown function or no hit found

SUPPLEMENTARY TABLE 12 | Gene ontology distribution at level 2 in two *M. oryzae* genomes.

Biological Process	GO-id	GO-term	RML-29 genes	RP-2421 genes
	GO:0009987	Cellular process	2174	2246
	GO:0008152	Metabolic process	1962	2405
	GO:0044699	Single-organism process	905	892
	GO:0051179	Localization	636	600
	GO:0071840	Cellular component organization or biogenesis	265	263
	GO:0050896	Response to stimulus	163	221
	GO:0023052	Signaling	159	177
	GO:0065007	Biological regulation	89	351
	GO:0000003	Reproduction	9	12
	GO:0032502	Developmental process	8	9
	GO:0051704	Multi-organism process	7	14
	GO:0040007	Growth	1	2
	GO:0022610	Biological adhesion	0	4
	GO:0002376	Immune system process	0	3
	GO:0040011	Locomotion	0	2
	GO:0048511	Rhythmic process	0	1
**Molecular Function**	GO:0003824	Catalytic activity	2988	2972
	GO:0005488	Binding	2673	2887
	GO:0005215	Transporter activity	320	265
	GO:0001071	Nucleic acid binding transcription factor activity	215	200
	GO:0005198	Structural molecule activity	135	139
	GO:0030234	Enzyme regulator activity	52	54
	GO:0060089	Molecular transducer activity	36	37
	GO:0000988	Protein binding transcription factor activity	19	15
	GO:0005085	Guanyl-nucleotide exchange factor activity	1	2
	GO:0016209	Antioxidant activity	0	12
	GO:0004872	Receptor activity	0	8
	GO:0009055	Electron carrier activity	0	1
**Cellular Component**	GO:0005623	Cell	1348	1331
	GO:0043226	Organelle	1073	1033
	GO:0016020	Membrane	1012	974
	GO:0032991	Macromolecular complex	134	247
	GO:0005576	Extracellular region	57	67
	GO:0031974	Membrane-enclosed lumen	35	54
	GO:0019012	Virion	0	1

SUPPLEMENTARY TABLE 13 | List of results obtained with different softwares used in secretome analysis.

Genome	RML-29	RP-2421
Predicted proteins	12746	12957
SignalP	1817	1839
TargetP	2550	2612
TMHMM	2194	2238
PredGPI-anchor	18	21
ProtComp	1133	1168
WolfPSort	669	675
Refined scretome	669 (344M + 325UK)	675 (347M + 328UK)

M: Matched with functionally characterized proteins; UK: Unknown or hypothetical type proteins

SUPPLEMENTARY TABLE 14 | Identification of repeats, SNPs, segmental duplications (SDs) and genes from *M. oryzae* strains.

Strain	Genome size (Mb)	Interspersed repeats (% of genome)	SNPs	SDs	Genes
**RML-29**	42.2	4656 (10.72)	157844	153	12746
**RP-2421**	44.8	6995 (11.03)	127677	181	12957
**70-15**	40.9	3738 (10.83)	-	218	12433
**P131**	37.7	2231 (1.90)	82587	71	11672
**Y34**	38.4	2226 (2.15)	97402	119	11790

- Not applicable

SUPPLEMENTARY TABLE 15 | Occurrence of SINE inter- or intra- genic regions and number of genes that contain the repeat.

Strain	SINE	Inter-genic	Intra-genic	Function known	Hypothetical	Unknown
**70-15**	146	28	118	45	71	2
**RML-29**	79	28	51	28	14	9
**RP-2421**	2029	1893	136	47	57	32
**P131**	29	9	20	9	11	0
**Y34**	25	9	16	8	8	0

SINE- Short Interspersed Nuclear Element

SUPPLEMENTARY TABLE 16 | Clusters of orthologs and paralogs identified from five *M. oryzae* genomes.

**Particulars**	**Number**
Total number of genes	61598
Total orthologous groups	11883
Total orthologous groups (5 genomes and at least one gene from each genome)	10291
Total orthologous groups, removed in-paralogs (5 genomes and 5 genes)	10013
Four genomes containing groups	636
Three genomes containing groups	463
Two genomes containing groups	470
Single genome containing groups	23

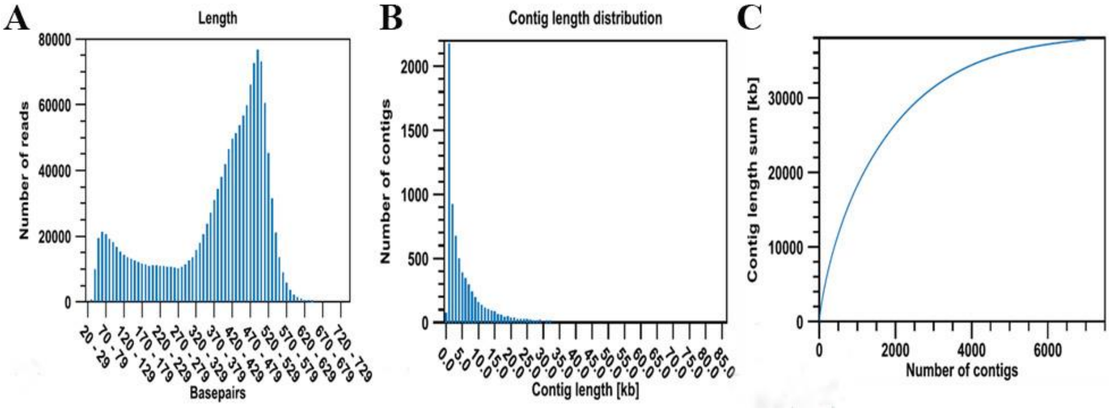

SUPPLEMENTARY FIGURE 1 | Distribution of reads and contigs of RML-29 genome. (**A**) Demonstrating length of reads before assembly, while after assembly, presenting (**B**) length and (**C**) number of contigs.

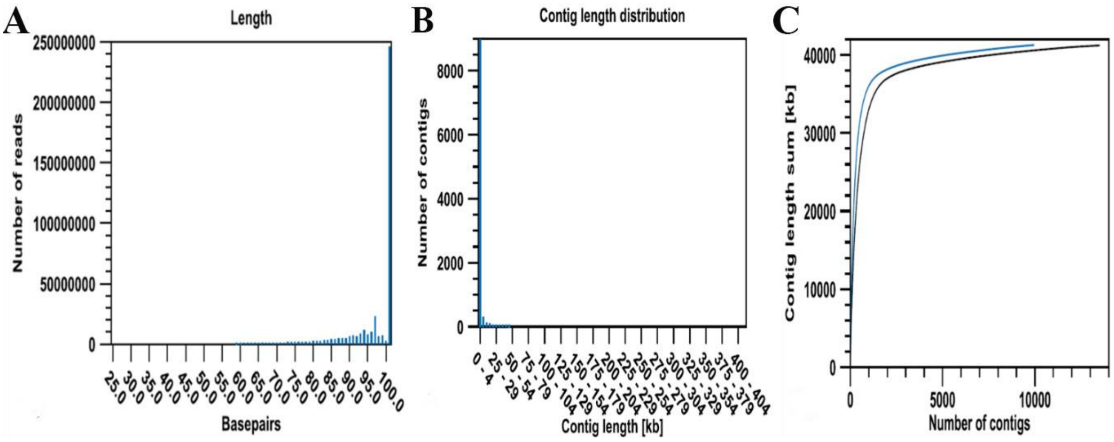

SUPPLEMENTARY FIGURE 2 | Distribution of reads and contigs of RP-2421 genome. (**A**) Showing length of reads before assembly, while after assembly length and number of contigs display in (**B**) and (**C**), respectively. Blue and grey lines in figure (**C**) is showing number of contigs with 'N' and without 'N' in the sequences, respectively.

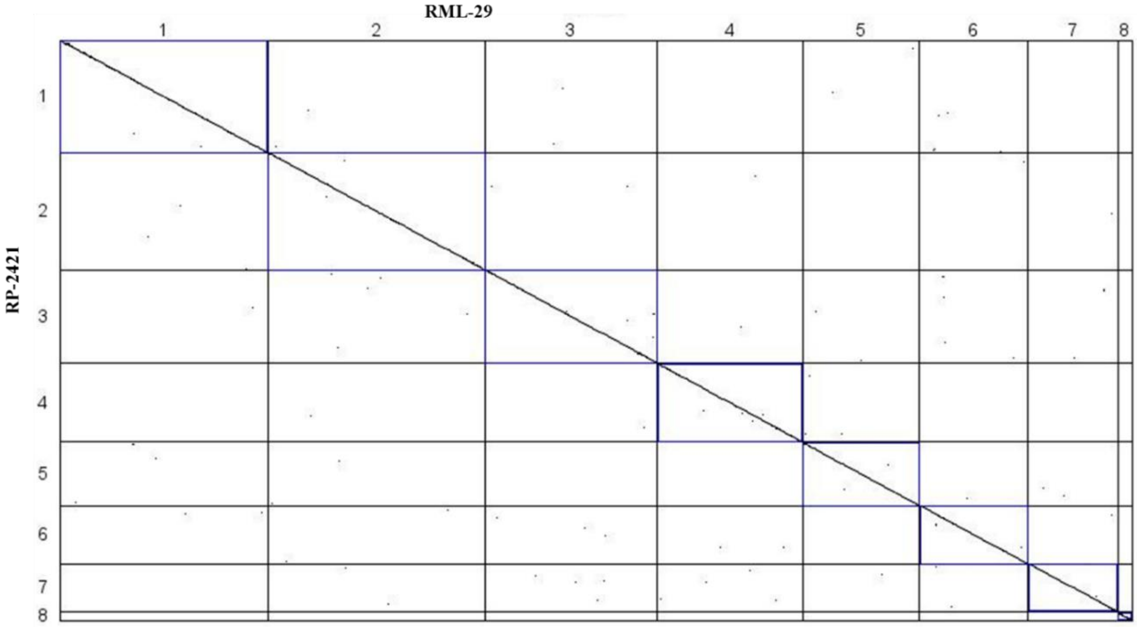

SUPPLEMENTARY FIGURE 3 | Dot plot comparison of RML-29 and RP-2421 genomes. Blue colored rectangles show size of respective chromosome.

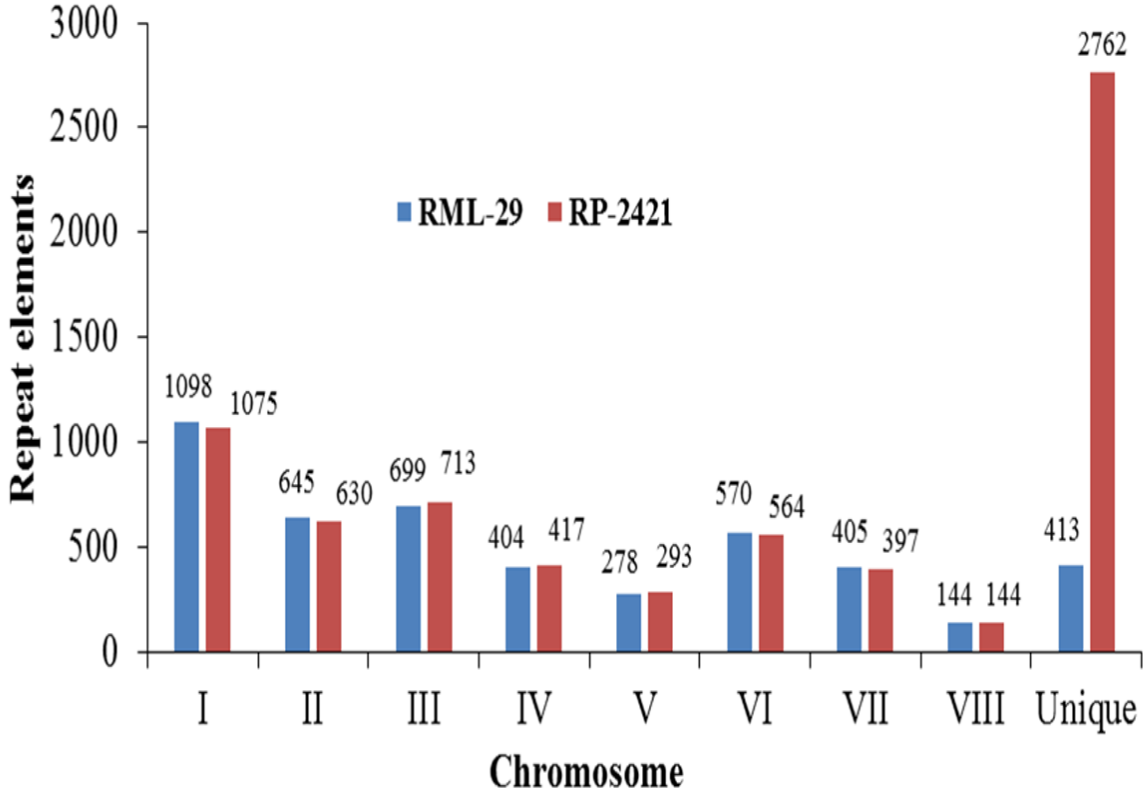

SUPPLEMENTARY FIGURE 4 | Chromosome wise distribution of repeat elements in two *M. oryzae* genomes.

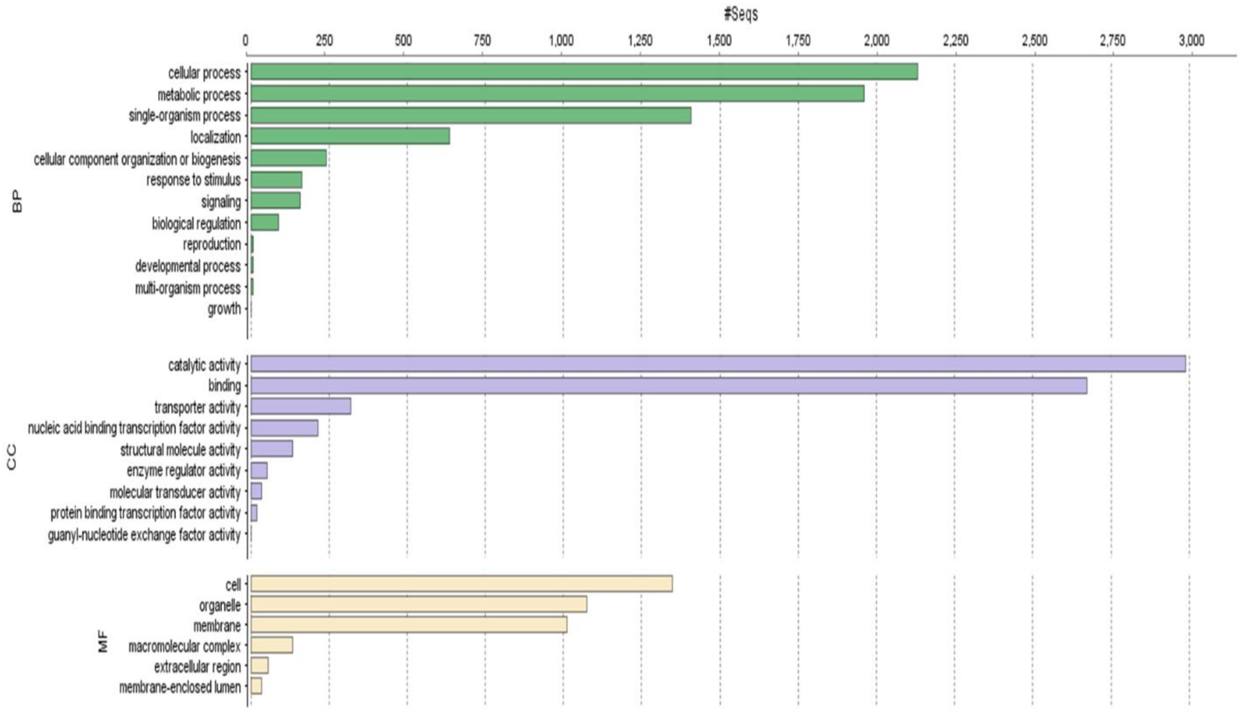

SUPPLEMENTARY FIGURE 5 | Sequence distribution per gene ontology (GO) of RML-29 genome. This distribution shows all the three major GO terms, biological process (BP), cellular component (CC) and molecular function (MF). Number of genes (sequences) given at the top of bar chart, while three major GO terms and their sub-terms present at left side of the chart.

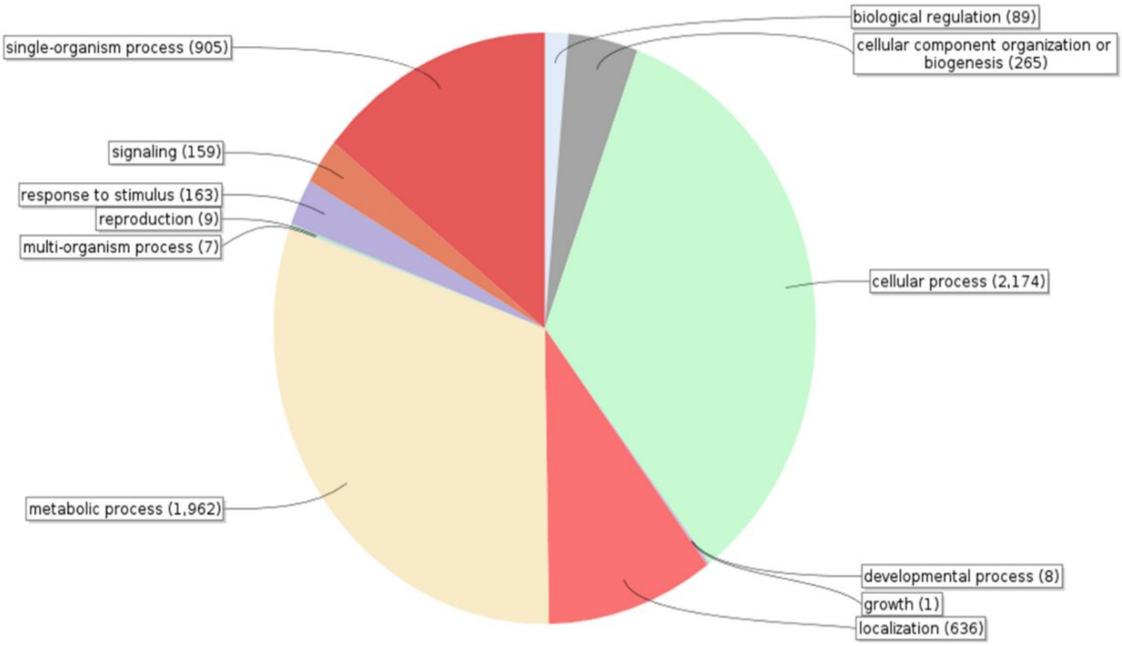

SUPPLEMENTARY FIGURE 6 | Gene ontology (GO) distribution of RML-29 genome at biological process level 2. GO terms of biological process and genes come under the terms indicate in rectangles and brackets, respectively.

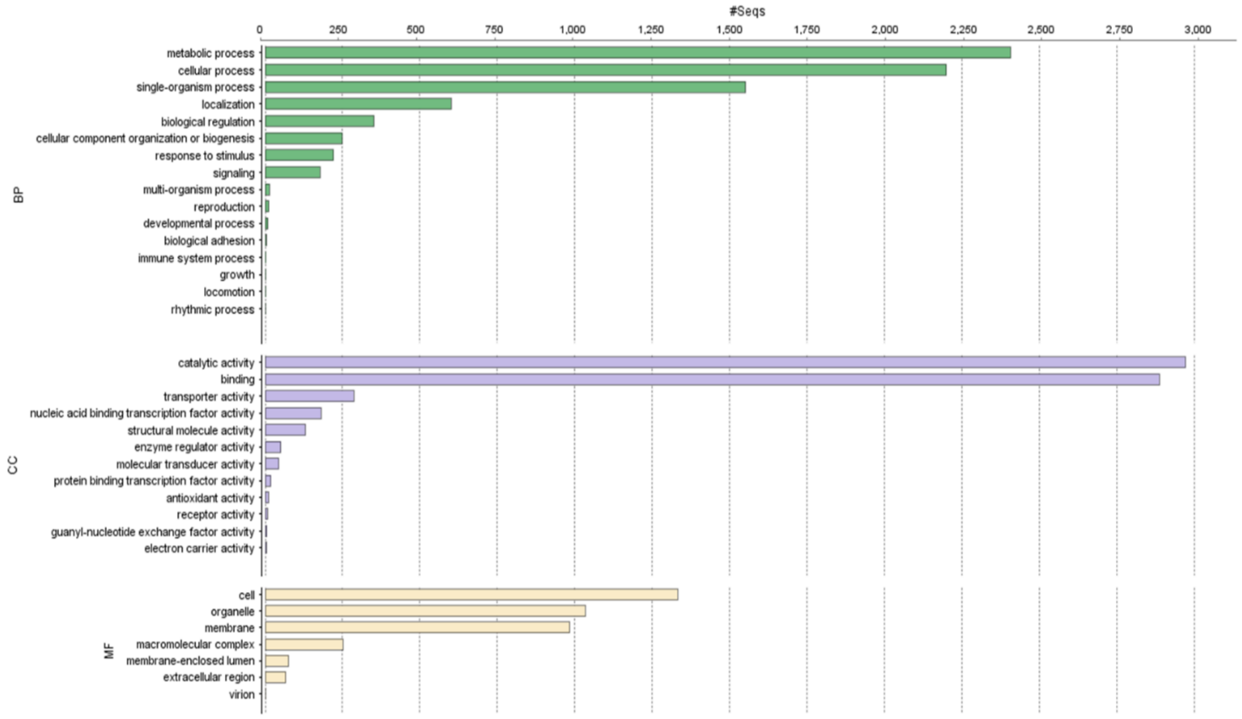

SUPPLEMENTARY FIGURE 7 | Sequence distribution per gene ontology (GO) of RP-2421 genome. This distribution shows all the three major GO terms, biological process (BP), cellular component (CC) and molecular function (MF). Number of genes (sequences) are at top of bar chart, while three major GO terms and their sub-terms present at left side of the chart.

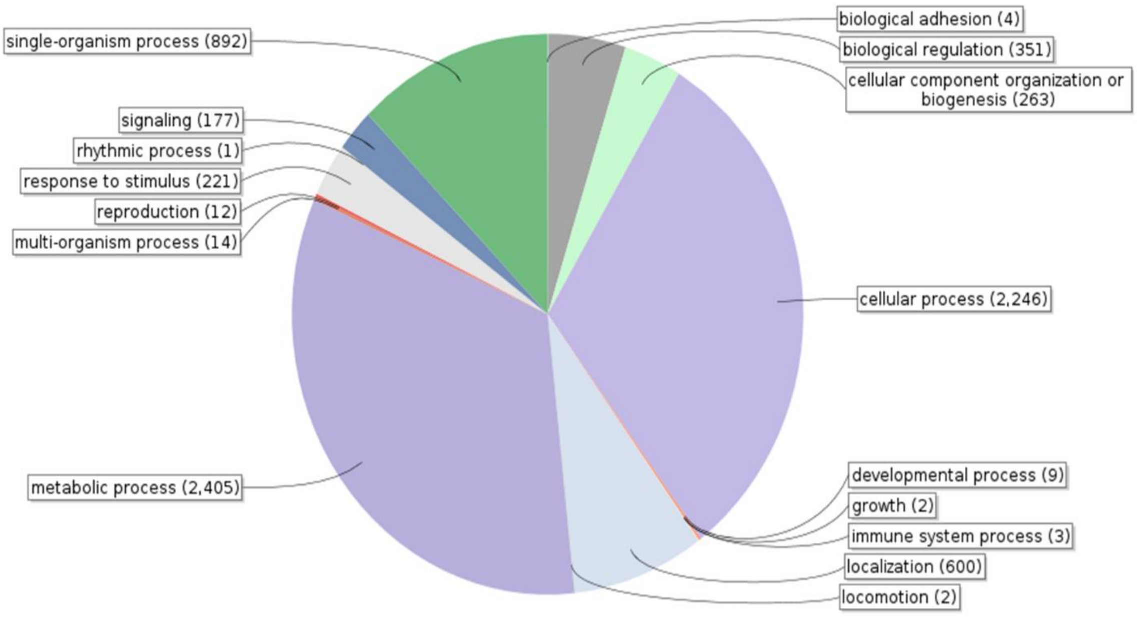

SUPPLEMENTARY FIGURE 8 | Gene ontology (GO) distribution of RP-2421 genome at biological process level 2. GO terms of biological process and genes come under the terms indicate in rectangles and brackets, respectively.

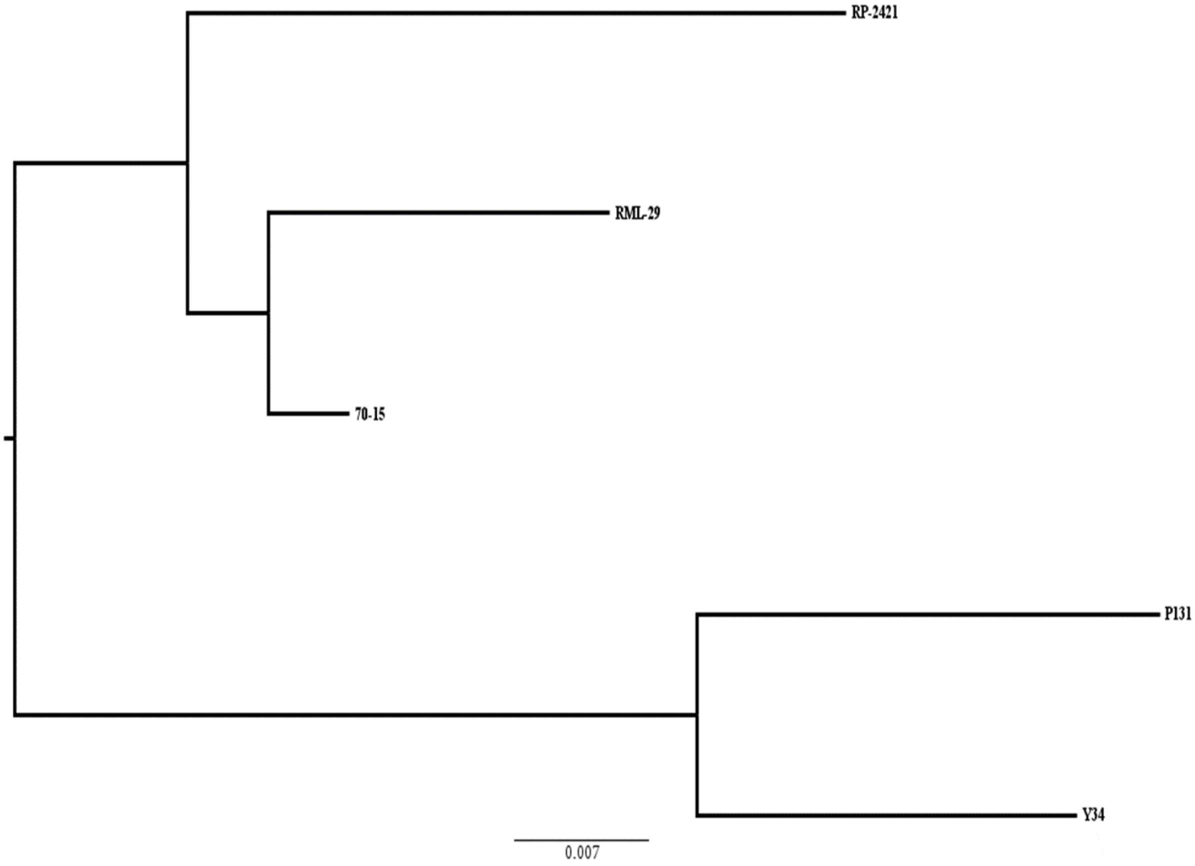

SUPPLEMENTARY FIGURE 9 | A phylogenetic tree of five *M. oryzae* isolates. Relatedness among the isolates based on the assembled whole genome sequences of these isolates. Scale of substitution rate per site is given at the bottom of the tree.
